# The glia of the adult *Drosophila* nervous system

**DOI:** 10.1002/glia.23115

**Published:** 2017-01-30

**Authors:** Malte C. Kremer, Christophe Jung, Sara Batelli, Gerald M. Rubin, Ulrike Gaul

**Affiliations:** ^1^ Gene Center and Department of BiochemistryCenter of Protein Science Munich (CIPSM), Ludwig‐Maximilians‐University MunichGermany; ^2^ Janelia Research Campus, Howard Hughes Medical InstituteHelix DriveAshburnVirginia

**Keywords:** glial subtypes, morphology, glial cell interaction, GAL4 lines, multicolor mosaic

## Abstract

Glia play crucial roles in the development and homeostasis of the nervous system. While the GLIA in the *Drosophila* embryo have been well characterized, their study in the adult nervous system has been limited. Here, we present a detailed description of the glia in the adult nervous system, based on the analysis of some 500 glial drivers we identified within a collection of synthetic GAL4 lines. We find that glia make up ∼10% of the cells in the nervous system and envelop all compartments of neurons (soma, dendrites, axons) as well as the nervous system as a whole. Our morphological analysis suggests a set of simple rules governing the morphogenesis of glia and their interactions with other cells. All glial subtypes minimize contact with their glial neighbors but maximize their contact with neurons and adapt their macromorphology and micromorphology to the neuronal entities they envelop. Finally, glial cells show no obvious spatial organization or registration with neuronal entities. Our detailed description of all glial subtypes and their regional specializations, together with the powerful genetic toolkit we provide, will facilitate the functional analysis of glia in the mature nervous system. GLIA 2017 GLIA 2017;65:606–638

## Introduction

Glia constitute the non‐neuronal population of cells in the nervous system. While originally thought of as inert “glue” (“glia” in Greek) that simply fills the space between neurons, research over the past decade suggests a richer and much more nuanced picture: In vertebrates and invertebrates, glia have been shown to perform a surprisingly wide range of functions in developing and mature nervous systems. During development, glia regulate neuronal cell numbers through control of neural stem cell proliferation, trophic support to neurons and elimination of dying neurons (for review see Corty and Freeman, 2013; Hidalgo et al., 2011; Liu et al., 2014). Glia also play a crucial role in establishing and modulating neuronal connectivity, from axon guidance, regulation of synaptic growth and plasticity, to effecting neuronal remodeling (for review see: Barres, 2008; Edenfeld et al., 2005; Oland and Tolbert, 2003; Stork et al., 2014).

In the mature nervous system, glia maintain ionic homeostasis, take up neurotransmitters after synaptic events, form and maintain the blood‐brain barrier, act as the main immune cells in the nervous system, and modulate synaptic activity as well as animal behavior (for review see: Barres, 2008; Corty and Freeman, 2013; Edenfeld et al., 2005; Featherstone, 2011; Haydon et al., 2009; Hindle and Bainton, 2014; Jackson, 2011; Oland and Tolbert, 2003; Stork et al., 2014; Zwarts et al., 2015). Recently, glia have been shown to play important roles in the aging of the nervous system including memory loss and neurodegeneration (Liu et al., 2015; Yamazaki et al., 2014). During evolution, the ratio between neurons and glia has changed in favor of the glia, with estimated numbers ranging from 15% in fly, 50% in mouse to 90% in humans, suggesting an expanding role of glia as the complexity of the nervous system increases.

*Drosophila* provides an excellent paradigm for studying the role of glia in the nervous system. *Drosophila* glia share crucial functional and anatomical features with their vertebrate counterparts (Freeman and Doherty, 2006; Stork et al., 2012). They engage in a mutually trophic relationship with neurons, provide neurotransmitter and ionic homeostasis, and serve as immune cells. Their morphologies include blood‐brain barrier forming epithelia, ensheathing, and astrocyte‐like cells. Importantly, *Drosophila* offers a highly developed, sophisticated genetic toolset for both morphological and functional analysis (Nern et al., 2011; Pfeiffer et al., 2010; Venken et al., 2011).

**Figure 1:**
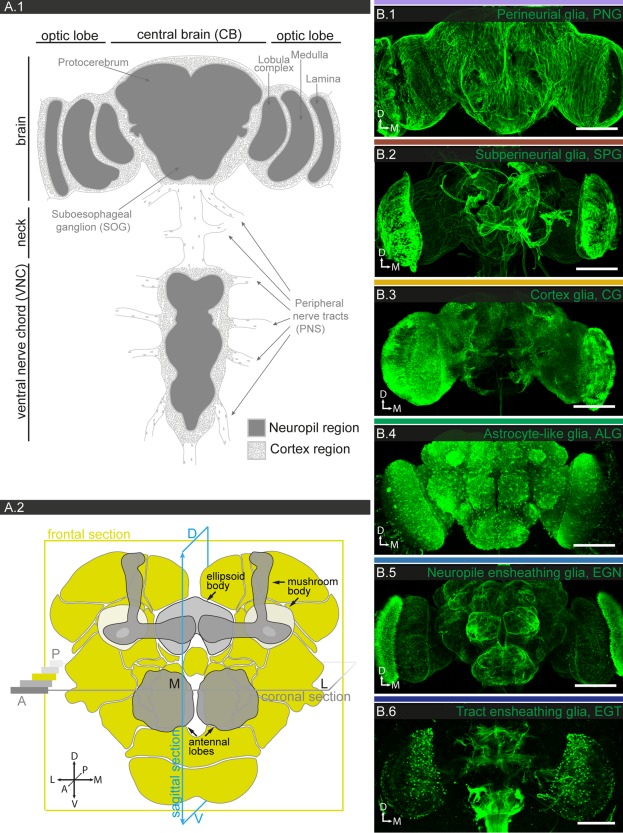
Anatomy of the adult *Drosophila* central nervous system and its generic glial cell types. **A.1**: Schematic of the central nervous system (CNS). The cortical regions (dotted areas) contain all neuronal and most glial cell bodies, while the neuropile regions (grey areas) contain the synaptic connections. **A.2**: Magnified schematic of the central brain. The different neuropile regions are colored (light green), regions of particular interest in this study are highlighted (shade of gray). The planes of the most frequently used confocal sections (frontal, sagittal, coronal) are illustrated, the orientation of the brain is depicted as a coordinate system (A: anterior; P: posterior; D: dorsal; V: ventral; M: medial; L: lateral). **B.1–6**: Different generic glial cell subtypes are visualized using subtype‐specific drivers and a membrane‐tagged reporter (UAS‐mCD8GFP); 130–160 μm projections are shown. Note the color code for the different glial subtypes **B.1**: PNG (lavender; 85G01‐Gal4), **B.2**: SPG (dark red; 54C07‐Gal4), **B.3**: CG (mustard; 77A03‐Gal4), **B.4**: ALG (green; 86E01‐Gal4), **B.5**: EGN (light blue; 56F03‐Gal4), and **B.6**: EGT (dark blue; 75H03‐Gal4). Scale bar = 100 μm in **B.1–6**.

In the *Drosophila* central nervous system, neuronal cell bodies are located in cortical regions, while synaptic connections are sequestered in the neuropiles; small and large axon tracts connect the different neuropiles (Fig. 1A.1; Meinertzhagen, 1993; Strausfeld, 1976). Peripheral nerves connect sensory organs and musculature with the central nervous system. Glia are found associated with all these anatomical structures. Previous studies have identified the generic subtypes of glia at different stages of development and in different regions of the nervous system (Awasaki et al., 2008; Doherty et al., 2009; Edwards and Meinertzhagen, 2010; Granderath and Klambt, 1999; Hartenstein, 2011; Ito et al., 1995; Klambt et al., 1991): Cortex glia (CG) in the cortical regions, astrocyte‐like (ALG) and ensheathing glia (EG) in the neuropile regions, ensheathing glia (EGN called wrapping glia in the PNS), associated with central axon tracts and peripheral nerves, and finally, two sheet‐like glia, subperineurial (SPG), and perineurial glia (PNG), which together form a contiguous surface that covers both central and peripheral nervous system. Over the years, the nomenclature has been converging on the terminology we use here, which is based on the location and/or morphology of the cells (Awasaki et al., 2008; Edwards and Meinertzhagen, 2010; Hartenstein, 2011). Our analysis of a large set of glial expression patterns reveals a strong correlation with morphology, which further supports this simple and consistent glial nomenclature.

Understanding the diversity of glial functions will require a thorough investigation of their full range of tasks in the nervous system. Whether the different glial subtypes perform specialized functions, and, to which extent glial functions in *Drosophila* compare with their vertebrate counterparts remain important open questions. However, a systematic genome‐wide analysis of glial function, especially in the adult, has been hampered by the lack of a comprehensive and detailed description of the glia in the mature nervous system and the development of appropriate tools for their genetic manipulation.

In this study, we begin to address these issues by investigating the morphologies of the different glial subtypes and their cellular interactions with both glial neighbors and neurons, taking advantage of a panel of glial‐specific GAL4 drivers we identified from the Janelia GAL4 collection (Jenett et al., 2012; Pfeiffer et al., 2008). Our examination of brain and ventral nerve chord identified morphologic features of the different glial subtypes and their interaction with glial neighbors; a more focused analysis of the visual and olfactory pathways revealed the interactions of glia with different neuronal compartments in these well‐studied brain areas.

## Materials and Methods

### Fly Strains and Genetics

The expression patterns of the Janelia GAL4 driver collection had previously been established using a membrane‐tagged GFP reporter (UAS‐mCD8‐GFP) and confocal imaging of entire adult brains (Jenett et al., 2012; Pfeiffer et al., 2008). By screening through the maximum density projections of this image collection, we identified lines with putative glial expression. For further analysis, we selected lines that showed specific expression in one or two glial subpopulations, no neuronal background, and limited mosaicism of expression. A complete annotated list of GAL4 lines with glial expression is available online (www.janelia.org/gal4-gen1). GAL4 strains are available through the Bloomington Stock Center.

Glial expression of drivers was ascertained using a nuclear GFP reporter (UAS‐nlsGFP) and colabeling with the pan‐glial marker REPO; these nuclear colabelings were also used to determine the cell number of specific glial subpopulations and the entire glial population in the adult brain. The morphology of individual cells and the relationship between neighboring cells was determined by multicolor mosaic experiments using the MCFO technique (Nern et al., 2015). In these experiments, a series of three differently tagged reporters under UAS control is kept silent by FRT‐flanked transcriptional terminators. Through a brief pulse of heat shock‐induced FLPase expression, terminators are removed randomly in individual cells, expression occurs only in cells that also express a (glial) GAL4 driver. This leads to a (sparse) patchwork of differently colored cells of a given glial cell type. To study the relationship between two different cell populations (glia/glia or glia/neuron), LexA versions of the best glial drivers were generated. The two binary expression systems, GAL4‐UAS (Brand and Perrimon, 1993; Pfeiffer et al., 2008) and LexA‐LexAOp (Lai and Lee, 2006; Pfeiffer et al., 2010), were then used to concurrently label two distinct cell populations with strong well‐defined reporters (UAS‐myr‐smGFP‐HA, LexAOp‐myr‐sm‐GFP‐V5; Pfeiffer et al., 2010; Tuthill et al., 2013). All drivers and reporters used in this study are listed in Table 2.

**Table 1:** Characterization of Generic Subtype‐Specific Drivers‐Expression Pattern and Cell Number

Driver	Generic subtype	Region	Additional drivers	Cell count (generic)	Cell count (compensation)	#	+/− (SEM)
R85G01‐Gal4	Perineurial	Entire CNS	–	2246	–	2246	41 (*n* = 5)
R54C07‐Gal4	Subperineurial	Entire CNS	–	300	–	300	15 (*n* = 12)
R54H02‐Gal4	Cortex	Not in lamina	53B07‐Gal4	1409	1226	2635	46 (*n* = 9)
			46H12‐Gal4				
R86E01‐Gal4	Astrocyte‐like	Mosaic in lamina	55B03‐Gal4	3668	950	4618	176 (*n* = 7)
R56F03‐Gal4	Neuropile ensheathing	No tracts	(75H03‐Gal4)	3106	*Next row*	*3106*	116 (*n* = 8)
R75H03‐Gal4	Tract ensheathing	Not in lamina	–	616	–	616	56 (*n* = 7)
**ALL**						**13523**	
**REPO‐positive**						**15703**	1717

Listed are the best drivers for each generic glial subtype. Expression may show mosaicism or missing in certain brain regions. In such cases, we used compensatory drivers, which are expressed exclusively in the missing/suboptimal region, for a complete cell count. In particular, no driver with expression in all ensheathing glia was recovered, and therefore complementary EGN and EGT drivers were used. Subtype cell count is thus the sum of generic plus compensatory cell count; the standard error of the mean, as well as the number of brains counted is indicated in the last column. The total number of glial cells within the adult brain and ventral nerve chord was estimated by counting REPO‐positive nuclei.

**Table 2:** Summary of Drivers, Reporters, and Antibodies used in this Study

Drivers						
General	Descriptor	Abbrev.	Specificity	Genotype	Source	Comment
*PNG*	Perineurial glia	PNG	Generic	R85G01‐GAL4 (attP2)	JFRC	
	Perineurial glia	PNG	Generic	R85G01‐LexA (attP40)	JFRC	
	Lamina perineurial glia	L‐PNG	Lamina	R47G01‐GAL4 (attP2)	JFRC	*Fenestrated glia*
	Lamina chalice glia	L‐cPNG	Lamina	R27H11‐GAL4 (attP2)	JFRC	
*SPG*	Subperineurial glia	SPG	Generic	R54C07‐GAL4 (attP2)	JFRC	
	Subperineurial glia	SPG	Generic	R54C07‐LexA (VK00027)	JFRC	
	Lamina subperineurial glia	L‐SPG	Lamina	R50A12‐GAL4 (attP2)	JFRC	*Pseudo‐cartridge glia*
*CG*	Cortex glia	CG	Generic	R77A03‐GAL4 (attP2)	JFRC	
	Cortex glia	CG	Generic	R77A03‐LexA (attP40)	JFRC	
	Cortex glia	CG	Generic	R54H02‐GAL4 (attP2)	JFRC	
	Lamina distal cortex glia	L‐dCG	Lamina	R53B07‐GAL4 (attP2)	JFRC	*Distal satellite glia*
	Lamina proximal cortex glia	L‐pCG	Lamina	R44B12‐GAL4 (attP2)	JFRC	*Proximal satellite glia*
	Lamina proximal cortex glia	L‐pCG	Lamina	R46H12‐GAL4 (attP2)	JFRC	*Proximal satellite glia*
*ALG*	Astrocyte‐like glia	ALG	Generic	R86E01‐GAL4 (attP2)	JFRC	
	Astrocyte‐like glia	ALG	Generic	R86E01‐LexA (VK00027)	JFRC	
	Lamina astrocyte‐like glia	L‐ALG	Lamina	R55B03‐GAL4 (attP2)	JFRC	*Epithelial glia*
*EG*	Neuropile ensheathing glia	EGN	Generic	R56F03‐GAL4 (attP2)	JFRC	
	Neuropile ensheathing glia	EGN	Generic	R56F03‐LexA (VK00027)	JFRC	
	Tract ensheathing glia	EGT	Generic	R75H03‐GAL4 (attP2)	JFRC	
	Lamina	L‐EGN	Lamina	R60F04‐GAL4 (attP2)	JFRC	*Marginal glia*
	Lamina	L‐EGN	Lamina	R35E04‐GAL4 (attP2)	JFRC	*Marginal glia*
	Chiasm glia	XGO/XGI	Chiasms	R53H12‐GAL4 (attP2)	JFRC	
	Chiasm glia	XGO/XGI	Chiasms	R53H12‐LexA (attP40)	JFRC	
*Neurons*	KC			17d‐Gal4	Manoli et al., 2005, Akalal et al., 2006	
	PNs	PN	Few cells	mz19‐Gal4	Bloomington	
	PNs	PN	Many cells	GH146‐Gal4	Bloomington	
**Reporters**	**Descriptor**	**Genotype**				
	Membrane tagged GFP	UAS‐mCD8‐GFP			JFRC	
	Membrane tagged GFP	UAS‐myr‐smGFP‐V5			JFRC	
	Nuclear GFP	UAS‐nlsGFP			Bloomington	
	Cytoplasmic GFP	UAS‐DM21‐GFP‐BP			JFRC	
	Double labeling	UAS‐myr‐smGFP‐HA; LexAop‐myr‐smGFP‐V5			JFRC	
	McFlip	HsFlipPestOpt; UAS‐Stop‐myr‐smGFP‐HA, UAS‐Stop‐myr‐smGFP‐V5, UAS‐Stop‐myr‐smGFP‐FLAG			JFRC	
**Antibodies**	**Descriptor**	**Target**	**Dilution**	**Host**	**Source**	
*Primary*	Neuropiles/synapses	NC82	1:25	Mouse	DSHB, Erich Buchner	
	Glial nuclei	REPO, 8D12	1:25	Mouse	DSHB, Corey Goodman	
	Neuronal nuclei	ELAV	1:10	Rat	DSHB, Gerald M. Rubin	
	Axon tracts	22C10	1:10	Mouse	DSHB, Seymour Benzer	
	Photoreceptor neurons	24B10	1:25	Mouse	DSHB, Seymour Benzer	
	Acetylcholine transporter	CHAT	1:25	Mouse	DSHB	
	GFP	GFP	1:500	Rabbit	Invitrogen	
	HA tag	HA	1:500	Rabbit	Cell signaling	
	FLAG tag	FLAG	1:100	Rat	Novus Biologicals	
*Conjugated*	V5‐tag:DyLight™‐549	V5	1:200	Mouse	AdSerotec	
	HA‐tag:DyLight™‐488	HA	1:200	Rabbit	Rockland	
*Secondary*	Pacific Blue	Mouse	1:200	Goat	Invitrogen	
	AlexaFluor 488	Rabbit	1:250	Goat	Invitrogen	
	DyLight 549	Mouse	1:200	Doneky	Jackson Laboratories	
	AlexaFluor 568	Rat	1:200	Goat	Invitrogen	
	DyLight 647	Rat	1:100	Donkey	Jackson Laboratories	
	DyLight 649	Mouse	1:100	Donkey	Jackson Laboratories	

Flies for the mosaic experiments were grown at 18°C, heat‐shocked 1 day after eclosion and dissected 2–3 days later. For all other experiments, flies were grown at 25°C and dissected 3–5 days after eclosion.

### Immunocytochemistry

Flies were anesthetized with CO_2_ to select the desired genotype, washed in cold 70% ethanol (30″), then cold PBS (30″) and kept in cold ExpressFive™ cell culture medium™ (Invitrogen) before and during preparation. Brains were dissected immediately using forceps and transferred into cold fixative. All subsequent steps were performed on a nutator at room temperature in 200 µL PCR tubes.

Brains were fixed in 2% paraformaldehyde (PFA, Electron Microscopy Sciences) in ExpressFive™ medium for 1 h. After three or more washes (15′ each) with adult brain washing solution [0.5%BSA (Sigma), 0.5% TX‐100 (Sigma) in PBS], the tissues were blocked with blocking solution [3% normal goat serum (Jackson Laboratories), 3% normal donkey serum (Jackson Laboratories), 0.5% TX‐100 in PBS] for 30′. Tissues were incubated with primary antibodies overnight, washed 3 × 1 h in adult brain washing solution, incubated with secondary antibodies overnight, washed 3 × 1 h in adult brain washing solution, followed by a final wash in PBS overnight. Tissues were mounted in VectaShield (Vector Laboratories) or 50:50 VectaShield and SlowFate™ Gold (Invitrogen).

Antibodies were obtained from the Developmental Studies Hybridoma Bank (DSHB), developed under the auspices of the NICHD, and maintained by The University of Iowa, Department of Biology, Iowa City, IA 52242, as well as commercial sources. All antibodies used in this study are listed in Table 2.

### Image Acquisition and Analysis

Stacks of serial confocal sections (0.32–1 μm) were acquired using a Zeiss LSM 710 with 20× (air), 40× (water), and 63× (oil) objectives. An automated table and MultiTime2010 (Zeiss) allowed us to scan multiple locations automatically, in which case 40× (with halogen‐free immersion medium, *n* = 1.33) and 63× (*n* = 1.52, oil) objectives were used. Processing of confocal stacks included maximum density projections, substack projections, orthogonal views, rotations, changes in channel hue and adjustment of brightness and contrast. Confocal images were processed with ImageJ (Abramoff et al., 2004; Rasband, 1997−2012); 3D reconstructions were created in Imaris (Bitplane). Counting of nuclei within confocal stacks was carried out as described in Supporting Information Figure S1 using a Definiens XD 2.0 based script. The structure density of astrocyte‐like glia (ALG) and synapses in different brain regions was determined and correlated as described in Supporting Information Figure S2 using a Definiens XD 2.0 based script. Figures and illustrations were prepared in Adobe Illustrator CS5 version 15.0.0.

## Results

### Annotation of Janelia Farm Gal4 Collection

We screened images generated from 6,650 GAL4 lines (Jenett et al., 2012) using the membrane‐tagged reporter UAS‐mCD8‐GFP, for drivers with glial expression in the adult brain. Our goal was two‐fold—to identify and annotate all lines with significant glial expression and to find, within this subset, lines that are well suited for both descriptive and functional analysis. Based on previously described glial morphologies (Awasaki et al., 2008; Doherty et al., 2009; Edwards and Meinertzhagen, 2010; Edwards et al., 2012), conspicuous location and non‐neuronal pattern, we identified 713 lines with potential glial expression. After validating their glial expression by co‐labeling with the glial‐specific marker REPO (Halter et al., 1995), we placed them into three main groups, depending on cell‐type specificity and uniformity of expression within a given cell type. The best 316 lines showed little or no mosaicism and no neuronal background expression: 79 had PNG and 47 had SPG expression, 23 had CG expression, 48 had astrocyte‐like and 58 had EG expression, and 46 had expression in more than one glial subtype. The remaining 379 lines displayed interesting glial patterns but were either severely mosaic or had strong neuronal expression and thus are not suitable for functional studies. We established a set of ∼100 reference lines that label the generic glial subtypes (Table 1) and region‐specific subpopulations of generic subtypes (Supp. Info. Table S1 and Fig. S3), and generated LexA versions for ∼50 of them. This set of reference lines provides the basis for the detailed study of glial anatomy in the adult fly brain presented here. For the five most frequently used Gal4 lines, we also characterized embryonic and larval expression (Supp. Info. Fig. S4), and compared their expression strength with *repo‐Gal4* in the locus and an 8 kb *repo* enhancer‐Gal4 fusion on the X (Supp. Info. Fig. S5); notably, all lines are stronger than the two *repo‐Gal4* drivers. The complete list of genetic tools, reporters, and antibodies used in our study is summarized in Table 2.

### Anatomical Analysis of Glia using the Reference Lines

Using this reference set of specific glial drivers, we generated a comprehensive description of glia in the adult (central) nervous system. Specifically, we assessed the number, location, and morphology of different glial subtypes, the (region‐specific) variation in cell size and shape within a given cell type and the physical relationship between cells of the same type. Glial expression of drivers was ascertained using a nuclear GFP reporter and colabeling with the pan‐glial marker REPO (Halter et al., 1995). These nuclear colabelings were also used to determine the cell number of specific glial subpopulations and the entire glial population in the adult brain; counting of nuclei was carried out using a custom Definiens script (see Materials and Methods). The morphology of individual cells and the relationship with neighboring cells was determined by a novel method for generating mosaic animals (multicolor flip‐out; MCFO) technique in which silenced reporters carrying different protein tags are randomly activated by short (hs) pulses of FLP recombinase (Nern et al., 2015). The tags are recognized with great sensitivity and thus afford visualization of cell morphology at unprecedented resolution. We investigated the relationship/interaction between different glial subtypes, and the relationship/interaction between glial and neuronal entities by employing two orthogonal expression systems (GAL4‐UAS, LexA‐LexAop) and highly sensitive reporters (Pfeiffer et al., 2010), as well as several neuron‐specific antibodies.

### Generic Glial Subtypes

#### Surface glia

The surface of the entire nervous system, including CNS and PNS, is covered by two thin glial layers, the PNG and SPG (Figs. 1B.1–2, 2, and 3). Developmentally, the SPG form the first contiguous layer during late embryogenesis and create the blood‐brain barrier through insulating intercellular septate junctions (Bainton et al., 2005; Schwabe et al., 2005). During larval stages, the PNG form a second layer on top of the SPG (Awasaki et al., 2008; Hartenstein, 2011; Stork et al., 2008). Similar to vertebrates, the invertebrate blood‐brain barrier plays a dual role by offering chemoprotection as well as selective transcellular transport of nutrients (Featherstone, 2011; Hindle and Bainton, 2014). Intriguingly, SPG have been recognized as a niche for neuronal stem cells whose proliferation they activate by local insulin signaling in response to the nutrient status of the animal (Chell and Brand, 2010; Speder and Brand, 2014).

#### Perineurial glia

**Figure 2:**
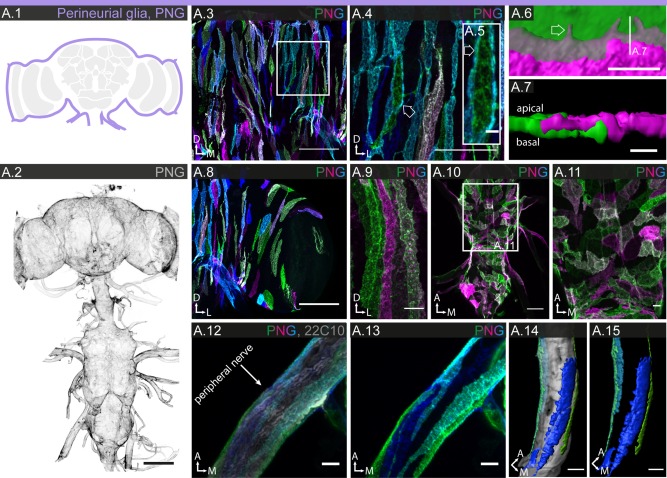
Morphology of PNG. **A.1**: Schematic showing the location of PNG (lavender) in the brain. **A.2**: PNG enclose the entire CNS and PNS; 85 μm projection. **A.3–15**: Individual cell morphologies of PNG in different regions of the CNS. **A.3**: In the central brain, PNG appear as long thin strips oriented along the dorso‐ventral axis; 61 μm projection. **A.4,5**: Higher magnification shows that PNGs interdigitate with neighboring cells through long thin extensions (arrows). **A.6,7**: 3D reconstruction of the cell boundary between two PNG showing small protrusions in the region of contact (arrow in **A.6**). As seen in cross section **(A.7)**, PNGs retain their relative proximo‐distal position along the entire length of contact. **A.8,9**: In the optic lobe, PNG appear as long thin strips along the dorso‐ventral axis; 20 µm projection. **A.10,11**: In the ventral nerve cord, PNG show a more varied morphology, ranging from elongated to square shaped, with orientation mostly along the medio‐lateral axis; 43 µm projection. **A.12,13**: In the PNS, PNG enclose the peripheral nerves; 43 µm projection. **A.14,15**: 3D reconstructions show that PNGs form narrow strips along the long axis of the nerve, without covering its entire circumference. Scale bar = 100 µm in **A.2**; 50 µm in **A.3**, **A.8**, **A.10**; 10 µm in **A.4**, **A.9**, **A.11–15**; 5 µm in **A.5**; 2 µm in **A.7**; 1 µm in **A.6**.

Using a reference line with uniform expression in all PNG (R85G01; Figs. 1B.1 and 2A.2), we count a total of ∼2250 PNG cells covering brain and ventral nerve chord, which is around 17% of the total glial cell population (Table 1). MCFO experiments reveal PNG as narrow, oblong cells (Figs. 2A.3–5, 8 and 9) with moderate variation in shape and orientation in different regions of the nervous system (Figs. 2A.3–5 and 2A.8–15). Some PNG lines show region‐specific expression (Supp. Info. Table S1 and Fig. S3), but only in the lamina is it accompanied by pronounced morphological specialization (Figs. 8 and 9). Overall, the PNG cover the entire nervous system in a densely tiled fashion. Interestingly, they achieve this by making two types of contacts. They form ledge‐like overlaps with the neighbors along their long axis (Fig. 2A.6–7) but also send out long thin extensions that brace their neighbors along their short axis (Fig. 2A.4–5).

#### Subperineurial glia

**Figure 3:**
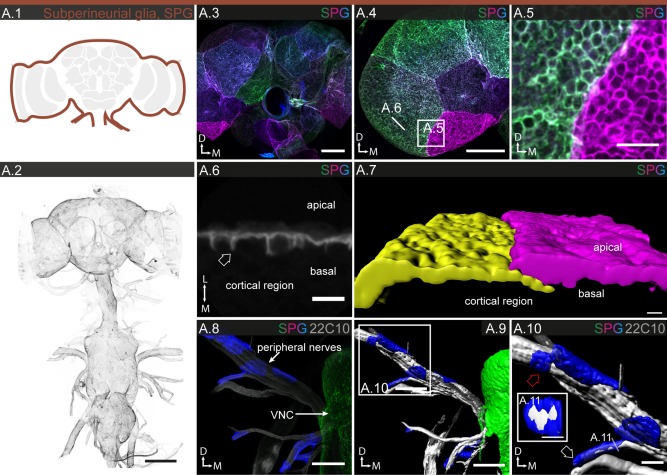
Morphology of SPG. **A.1**: Schematic showing the localization of SPG (dark red) in the brain. **A.2**: SPG enclose the entire CNS and PNS; 85 μm projection. **A.3–11**: Individual cell morphologies of SPG in different regions of the CNS and PNS. **A.3,4**: In the central brain (**A.3**) and optic lobe (**A.4**), SPG are thin, very large square‐shaped cells; 38 μm projection. **A.5,6** In higher magnification, many ring‐like membranous structures are apparent, which in cross section (**A.6**), reveal themselves as cap‐like basal protrusions (arrow) that cover individual neuronal cell bodies, which are also enveloped by CG, within the cortex. **A.7**: 3D reconstruction showing the overlap between two neighboring SPGs. **A.8–11**: Individual SPGs covering peripheral nerve; 102 µm projection. Cells have a square to oblong shape (red arrow in **A.10**) that extends along the long axis of the nerve; thinner nerves can be completely enveloped by a single cell (white arrow in **A.10**) forming a tube, as revealed by a cross section in **A.11**. **A.9,10** 3D reconstructions of **A.8** at two different magnifications. Scale bar = 100 µm in **A.2**; 50 µm in **A.3,4**, **A.8,9**; 10 µm in **A.5**; 5 µm in **A.6**, **A.10,11**; 1 µm in **A.7**.

Using a reference line with uniform expression in all SPG (R54C07, Figs. 1B.2 and 3A.2), we count a total of ∼300 SPG cells covering brain and ventral nerve chord, which is around 2% of the total glial population (Table 1). The MCFO experiments reveal SPG as large, nearly square‐shaped cells, with moderate variation in size and little variation in shape between different regions of the CNS (Fig. 3A.3–5). In the PNS, SPG can form elongated tubes around small peripheral nerves (Fig. 3A.10), but enclose larger nerves jointly with other cells (Figs. 3A.8–10; Supp. Info. Fig. S6). Again, while some of the SPG lines show region‐specific expression (Supp. Info. Table S1 and Fig. S3), only in the lamina is it accompanied by pronounced morphological specialization (Figs. 8 and 9). Altogether, the SPG form a densely tiled and tight epithelium (Fig. 3A.2) with moderate overlap between neighboring cells (Fig. 3A.7). The many ring‐like membranous structures within the cells (Fig. 3A.5) reveal themselves in cross section as cap‐like basal protrusions that cover individual neuronal cell bodies within the cortex (Fig. 3A.6). Note that, in addition, the neuronal cell bodies are also enveloped by a thin layer of CG (see below).

Together, the PNG and SPG form a 2–3 µm thick double layer that contiguously covers CNS and PNS in their entirety (Fig. 7A.2–4). While both glial subtypes form continuous sheets, their strategies differ vastly in terms of cell number, size, shape, and intercellular contact. During the massive growth of the nervous system and increase in surface area from embryo to adult, SPG retain their small number and massively increase their individual cell size by endoreplication (Unhavaithaya and Orr‐Weaver, 2012), while PNG (exponentially) grow in number by extensive cell division (Avet‐Rochex et al., 2012; Awasaki et al., 2008). The differences in the organization of the two cell layers may reflect a division of labor in generating the blood‐brain barrier, with the SPG creating the paracellular barrier and the PNG providing physical support. The tasks of chemoprotection and selective transport are likely shared between the two cell layers.

#### Cortex glia

The cortical regions of the CNS are populated by a single glial subtype, the CG (Figs. 1B.3 and 4), which proliferate during larval stages. In the adult, they encapsulate neuronal cell bodies and wrap neuronal processes as they cross the cortex region before entering the neuropile (Awasaki et al., 2008; Hoyle, 1986; Pereanu et al., 2005; Spindler and Hartenstein, 2010). CG have been suggested to provide trophic support to neurons (Stork et al., 2012), but so far little is known about their function. Interestingly, CG have been shown to exhibit activity‐dependent calcium oscillations and to regulate seizure susceptibility (Melom and Littleton, 2013).

**Figure 4:**
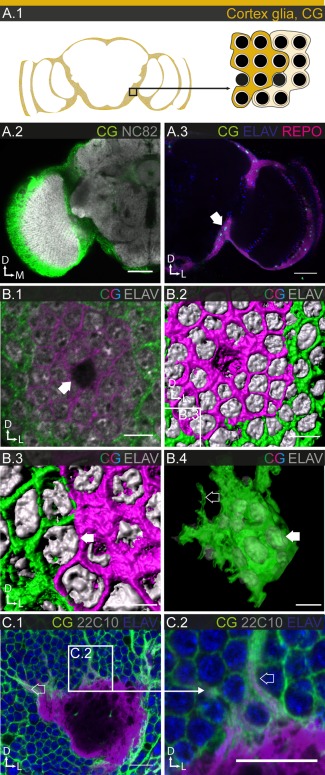
Morphology of CG. **A.1**: Schematic showing the location of CG (mustard) in the brain (left), and the relationship between individual CG and neuronal cell bodies (right). **A.2**: CG fill the cortical regions of the brain without entering the neuropile regions; single section. **A.3**: CG cell bodies are found exclusively in the cortical regions of the brain (arrow); single section. **B.1–4**: Individual cell morphologies of CG in the central brain. Single section (**B.1**) and 3D reconstruction (**B.2,3**) of the same cell (arrow in **B.1** points to glial nucleus), showing that a single CG cell envelopes 20–100 neuronal cell bodies. Neuronal cell bodies are wrapped individually, but two neighboring CG can contribute to wrapping one cell body (arrow in **B.3**). **B.4**: 3D reconstruction of an entire CG, with translucent membrane to reveal neuronal cell bodies within (filled arrow) and lamellipodial extensions (open arrow); 32 µm projection. **C.1,2**: Interaction of CG with neuronal cell bodies and fiber tracts; single section. CG support neuronal fibers (arrows) until they leave the cortical region. Scale = 50 µm in **A.2,3**; 10 µm in **B.1,2**, **C.1,2**; 10 µm in **B.3,4**.

Based on its stable and strong expression in most CG, we picked R54H02 as a reference line (Fig. 1B.3). However, since this line shows no expression in the lamina cortex, we used R53B07 and R46H12 as reference lines for that specific subpopulation. Using these reference lines together, we count a total of ∼2,600 CG cells, around 20% of the entire glial population (Table 1). Their nuclei are exclusively located in the cortical regions of the CNS (Fig. 4A.3). Again, while some of the CG lines show region‐specific expression (Supp. Info. Table S1 and Fig. S3), only in the lamina is it accompanied by pronounced morphological specialization (Figs. 8 and 9; Edwards et al., 2012; Saint Marie and Carlson, 1983a).

MCFO experiments show CG as large honeycomb‐like structures, in which neuronal cell bodies are enveloped individually. The number of enveloped cell bodies varies from a few (lamina; Fig. 9C.2) up to 100 (central brain; Fig. 4B.1). Neighboring CG abut with little overlap (Fig. 4B.2–3). 3D reconstruction of CG reveal an overall globular shape with fine lamellae protruding into surrounding areas (Fig. 4B.4), suggesting that CG minimize the area of contact with each other, while maximizing their contact with neuronal cell bodies. Apart from enveloping neuronal cell bodies, CG also wrap neuronal processes as they travel across the cortex (Fig. 4C.1–2). Interestingly, while recognizing neuronal surfaces in general, the CG do not seem to recognize individual neuronal units, such as cell bodies. We therefore often find that the enclosure for one neuronal cell body is created jointly by two neighboring CG (Fig. 4B.2–3).

#### Astrocyte‐like glia

**Figure 5:**
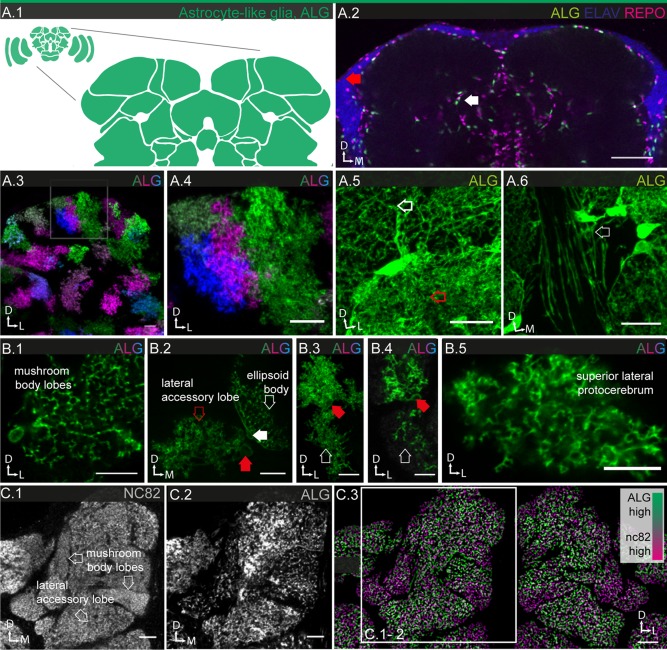
Morphology of ALG. **A.1**: Schematic showing the location of ALG (green) in the brain. **A.2**: Location of neuronal cell bodies is always in the cortical regions (red arrow), while ALG cell bodies are frequently found in between neuropile regions (white arrow). **A.3,4**: Individual cell morphologies of ALG in the central brain. ALG show variable size and morphologies but cover largely non‐overlapping areas; single sections. **A.5,6**: Higher magnification views of ALG morphology; 3 μm projections. **A.5**: The glial mesh can be sparse (white arrow) or dense (red arrow), but individual cells cannot be distinguished when all cells are labeled. **A.6**: ALG project fine but long processes (arrow) from the neuropile into tract regions. **B.1–5**: Individual ALG cell morphologies in the central brain; filled arrows point to glial cell bodies, open arrows to processes. **B.1**: Single cell with low density of processes projecting into two neuropile (sub)‐regions; single section. **B.2**: Two neighboring cells, projecting into different neuropiles, each showing a markedly different density of processes; 2 µm projection. **B.3,4**: A single cell, projecting into two different neighboring neuropiles with different density of processes; **B.3**: 40 µm projection, **B.4**: single section. **B.5**: Single cell with high density processes; single section. **C.1,2**: Synaptic **(C.1)** and ALG **(C.2)** densities in neuropiles of the protocerebrum; single sections. **C.3** The stainings shown in **C.1,2**, converted into structural density maps based on the distance between objects within a brain region and superimposed, show the anticorrelation of synaptic and glial densities. Regions with high synaptic density appear magenta, regions with high ALG density appear green (for description of algorithm see Materials and Methods; Supp. Info. Fig. S4). Scale bar = 50 µm in **A.2**; 10 µm in **A.3–6**, **B.1**,**2**,**5**, **C.1–3**; 5 µm in **B.3,4**.

ALG populate the neuropile regions (Fig. 5A.1), together with the EG discussed in the following section. Both cell types play an important role in ionic and neurotransmitter homeostasis (Edwards et al., 2012; Stork et al., 2012, 2014). Based on its stable and strong expression in most ALG, we picked R86E01 as a reference line (Fig. 1B.4). However, this line shows mosaic expression in the lamina and we therefore used R55B03 as a reference line for that specific subpopulation. Using the two reference lines together, we count a total of ∼4,600 ALG cells, around 34% of the total glial cell population (Table 1). In contrast to CG, the nuclei of ALG are not only found at the interface between cortical regions and neuropiles, but also between neuropile regions deep inside the brain (Fig. 5A.2; Awasaki et al., 2008).

Within the neuropile, ALG elaborate a dense meshwork comprised of a highly ramified cellular morphology, formed by processes of varying thickness (Fig. 5A.5), but in addition also project long filopodia into adjacent tract regions (Fig. 5A.6). MCFO experiments show that ALG vary in size and shape (Fig. 5A.3–4). Individual cells show the same tiling observed in the other glial subtypes; at the boundaries between two cells, their processes show modest overlap. Interestingly, ALG do not respect neuropile boundaries and often project two independent branches into two different neuropile regions (Fig. 5B.3–4), suggesting that, similar to CG, they are not dedicated to specific synaptic units such as glomeruli, layers or columns.

At higher magnification, the mosaics reveal that astrocytic processes vary strongly in their structural density—from wiry thin to thick—as a function of the specific neuropile the glia invade (Fig. 5B.1, 5). Even with two neighboring glial cells, one may form thin, the other thick processes as each invades a different neuropile (Fig. 5B.2). Even more astonishingly, as two branches of the same glial cell invade different neuropiles one may show low, the other high structural density (Fig. 5B.3–4).

We sought to further investigate this phenomenon that the structural density of ALG processes is indeed a neuropile‐specific feature and whether it may in fact be anti‐correlated with the synaptic density of the respective neuropile region. Lower magnification views of central brains double‐labeled with synapse‐ and astrocyte‐like specific markers support these observations. For instance, the mushroom body has a high density of synapses and shows a low structural density of astrocyte‐like processes. In contrast, the antennal and lateral accessory lobe as well as the optic tubercle have lower densities of synapses and show moderate to high structural densities of astrocyte‐like processes. In order to obtain an objective measure, we developed a custom Definiens algorithm (Supp. Info. Fig. S2) to quantify both densities in confocal sections (Fig. 5C.1–2). Super‐imposition of the density maps of astrocyte‐like processes and of synaptic areas reveal systemic anticorrelation (Fig. 5C.3). Given the important function of ALG in neurotransmitter homeostasis, this result is unexpected. Altogether, our findings suggest that the micromorphology of ALG is induced and determined by the density of synapses present in the neuropile they invade.

#### Ensheathing glia

EG are the second glial subtype found in neuropiles. However, they also associate with axon tracts (Figs. 1B.5–6 and 6A.1–3, B.1, C.1) and thus show a broader range of morphologies than other glial subtypes (Hartenstein, 2011). In the PNS, wrapping glia serve a homologous function by ensheathing peripheral nerves. Ensheathing (wrapping) glia have been shown to phagocytose neuronal debris after axonal injury and during normal synaptic growth (Corty and Freeman, 2013; Stork et al., 2012).

**Figure 6:**
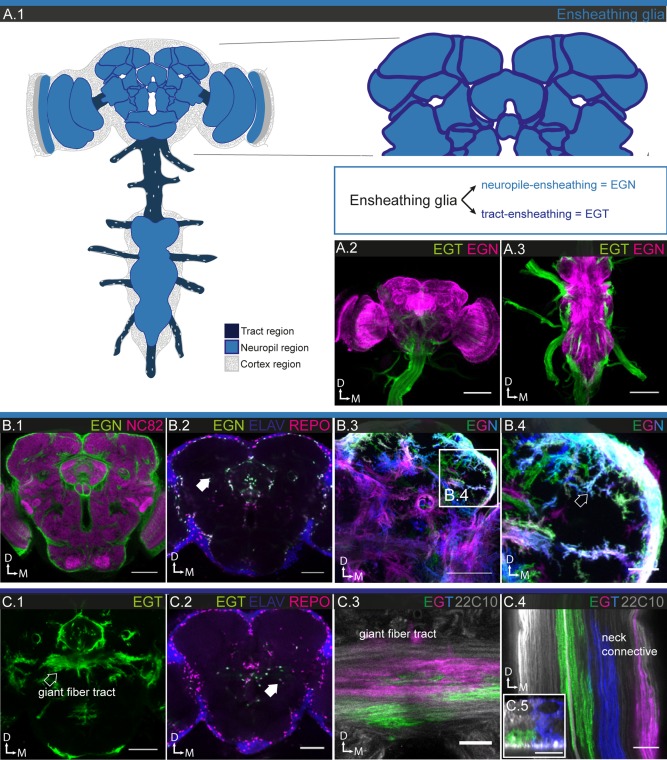
Morphology of ensheathing glia. **A.1**: Schematic showing the location of ensheathing glia (EG) in the nervous system, comprising two subsets, EGN (light blue) and EGT (dark blue). **A.2,3**: Double labeling of EGN and EGT, visualizing the entire EG population of the nervous system, 122 µm projections; **A.2**: brain and neck commissure; **A.3**: ventral nerve cord and PNS. **B.1–4**: EGN; **B.1**: general expression pattern in the central brain; single section; **B.2**: EGN cell bodies (arrow) are mostly found in between neuropile regions in the central brain; single section. **B.3,4**: Individual EGN cell morphologies in the protocerebrum; 25 µm projections. The EGN take on many different shapes and sizes, as they ensheath and project complex and fine protrusions (arrow) into the neuropile regions. **C.1–4** EGT; **C.1**: general expression pattern in the central brain; single section; **C.2**: EGT cell bodies (arrow) are mostly found in between neuropile regions in the central brain; single section. **C.3,4**: Individual EGT cell morphologies in giant fiber tract (**C.3)** and neck connective **(C.4);** single sections. EGT are extended along the long axis of the nerves; their ends are ragged but with their lateral neighbors they form a neatly contiguous sheath. Cross section shows that nerve tracts are completely ensheathed (**C.5**). Scale bar = 100 µm in **A.2,3**; 50 µm in **B.1–3**, **C.1**; 10 µm in **B.4, C.3–5**.

EG expression is observed at an intermediate frequency (58 out of 316); however, none of the GAL4 drivers are expressed in all EG. Most lines are expressed in glia that ensheath both neuropiles and tracts in varying proportions; much fewer are specifically expressed in EGT or EGN. To account for this problem, we picked two reference lines: R75H03, which drives expression in all tract, but no EGN, and R56F03, which drives expression in most neuropile and only very few tract ensheathing glia (EGT). Using these two reference lines, we estimate the number of EG in tracts at ∼600, in neuropiles at ∼3,100, and thus ∼3,700 altogether, which represents around 27% of the total glial population (Table 1). The finding that the majority of GAL4 lines are expressed in both EGN and EGT suggests a strong underlying communality between the two cell populations, conversely, the fact that expression is segregated in some lines lends support to the anatomically based distinction between the two. Many of the drivers expressed in EGT in the CNS are also expressed in glia wrapping nerves in the PNS (Fig. 6A.3), further supporting the notion that wrapping and EG belong to the same glial subtype.

The nuclei of the EGN are localized at the boundaries of all neuropiles, not only at the distal boundaries adjacent to the cortex regions but also at the proximal boundaries deeper in the brain that are abutting other neuropiles (Fig. 6B.2). MCFO experiments show that the EG form a dense layer around the neuropile regions. In addition, they frequently branch into the neuropiles first perpendicular and then parallel to their surface, with lamellae protruding at the outskirts (Fig. 6B.3–4). When entering the neuropiles, they mostly accompany neuronal projections, but occasionally also tracheal branches (see Fig. 10; Pereanu et al., 2007). Neighboring EG show little if any overlap (Fig. 6B.3–4).

The nuclei of the EGT are found in noncortical regions deep inside the brain (Fig. 6C.2) and along the neuronal tracts with which they associate. EGT envelop neuronal projections that connect different neuropiles and brain regions, such as the giant fiber tract (Fig. 4C.3), the neck connective (Fig. 4C.4) and peripheral nerves. The EGT form flat, long parallel strands that are frayed at the margins. Neighboring cells can interweave without much direct cell‐cell contact (Fig. 6C.3). Cross sections reveal that the glia envelope multiple fiber tracts (Fig. 6C.5).

Taken together, the principal morphology of all forms of EG is that of a sheet or a lamella, which then bends or folds up into tubes, covering surfaces of all shapes and forms.

### Interactions between the Different Glial Subtypes

**Figure 7:**
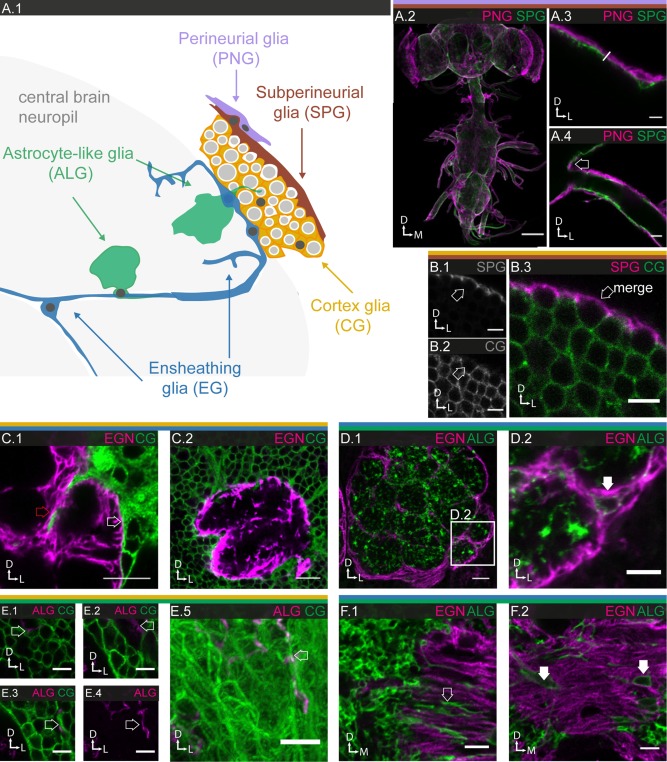
Characterization of glia‐glia interactions. **A.1**: Schematic depicting glial subtypes and their relationship to the different neuronal compartments. **A.2–4**: Relationship between PNG and SPG on the surface of nervous system; 85 µm projections. **A.3,4**: Higher magnification shows that the PNG lie directly on top of the SPG. The surface glial sheath is ∼2–3 µm thick and continues across the CNS‐PNS boundary, as seen at the exit of peripheral nerves (arrow in **A.4**). **B.1—3**: Relationship between SPG (**B.1**) and CG (**B.2**), merge in (**B.3**); central brain; single section. SPG (**B.1**) form a contiguous cover over the entire cortical region. Occasionally, CG do not fully envelop neuronal cell bodies, in which cases SPG provide the necessary closure (arrow). **C.1,2** Relationship between CG and EGN antennal lobe (**C.1**), mushroom body calyx (**C.2**); single section. The two subtypes abut in a very smooth fashion with little or no intermingling at the interface (white arrow). Occasionally, CG protrusions reach into neuropile regions, shown here a sub‐compartmental boundary within the antennal lobe (red arrow). **D.1,2**: ALG cell bodies (arrow) are embedded within the EGN layer; single section. **E.1–5**: Relationship between CG and ALG in cortical regions; **E.1–3**: single sections; **E.4,5**: 14 µm projections. ALG send fine processes into the CG region (arrows). **F.1,2**: Relationship between ALG and EGN, single sections. **F.1**: ALG processes (arrow) projecting into the EGN layer of a neuropile region in the protocerebrum. **F.2**: ALG cell bodies (arrows) are embedded in EGN sheath. Scale bar = 100 µm in **A.1**; 10 µm in **C.2**; 5 µm in **A.2,3, B.1–3, C.1, D.1–5, E.1–4**.

From our investigations of homotypic interactions between cells of the same glial subtype, certain general features begin to emerge. On the whole, the glia show tiling, that is, they minimize contact with their glial neighbors, while maximizing contact with the enveloped neuronal compartment (cell body, axons, dendrites, synapses) or larger neuronal units (neuropiles, columns, glomeruli). PNG and SPG form tight epithelia (Fig. 7A.2–4) with well‐defined areas of physical contact; however, the remaining subtypes show little overlap with their neighbors except for filopodial or lamellipodial extensions. Finally, glial cells seem to lack registration with the neuronal units they envelop and thus frequently share the task with their immediate glial neighbors.

Interestingly, similar features are also observed in the interaction between glia of different subtypes. On the outer surface of the cortex, CG encounter SPG. Occasionally, CG fail to envelop the distal‐most segment of neuronal cell bodies. In these cases, SPG complete the enclosure (Fig. 7B.1–3). On the inner surface of the cortex, CG encounter both ensheathing and ALG. Cortex and EG abut and form smooth boundaries with little overlap between the two cell types (Fig. 7C.1–2). However, CG wrap neuronal projections in the cortex region, a task that is taken over by the EG once the projections reach the cortex/neuropile border. ALG cell bodies are enclosed by EG at the margins of the neuropiles (Fig. 7D.1–2 and F.2). However, ALG send processes not only into the neuropile sheath, but also long filopodia into tracts (Fig. 7F.1) and even cortical regions (Fig. 7E.1–5).

EG help define boundaries of sub‐compartments (Fig. 7D.3) in many neuropiles, but neither ensheathing nor ALG are confined to specific neuronal units. More generally, astrocyte‐like and EG jointly populate the neuropiles but without any obvious spatial organization or registration with neuronal entities. The following sections, in which we describe the visual and olfactory systems, provide specific examples supporting these general observations.

### Glia in the Visual System

As a next step, we sought to study the interaction between glia and neurons in more detail. For this purpose, the visual system is particularly amenable. It makes up a large portion of the fly brain and, for decades, has been subject of extensive neuroanatomical and functional studies (for review Borst, 2009; Borst et al., 2010; Fischbach, 1989; Maisak et al., 2013; Meinertzhagen, 1993; Morante and Desplan, 2008; Takemura et al., 2013). The entire system is organized in a highly stereotyped, retinotopic fashion and consists of four large neuropiles—lamina, medulla, lobula, and lobula plate—that are connected by two large chiasms. In going from lower to higher order centers, visual information is first processed in a point‐by‐point fashion by columnar units of neuronal connectivity, which then becomes more integrated across the visual field/retina, leading to a more and more horizontal neuronal connectivity (Morante and Desplan, 2008; Sanes and Zipursky, 2010). The fact that each neuropile and chiasm has a highly reiterative but distinct organization lends itself to identifying and comparing features of glia‐neuron interactions in different scenarios. Finally, the glia found in the first neuropile of the visual system, the lamina, show strong regional specialization compared with other regions of the brain, which we sought to investigate in more detail.

### The Lamina

The lamina is the distal‐most optic neuropile and the first relay station in which photoreceptor axons terminate in a retinotopic fashion. Incoming photoreceptor axon bundles from ∼800 ommatidia connect to ∼800 lamina columns, in which complex synaptic connections are made between six photoreceptors (R1‐R6), five lamina output neurons (L1‐L5; Fig. 9A.2) and additional higher order neurons (Fischbach, 1989; Meinertzhagen, 1993; Rivera‐Alba et al., 2011; Tuthill et al., 2013).

Previous studies in *Musca* and *Drosophila* have identified six types of glial cells, which are organized in distinct layers: fenestrated, pseudo‐cartridge, distal and proximal satellite, epithelial and marginal glia (Edwards et al., 2012; Saint Marie and Carlson, 1983a, 1983b), for review: Edwards and Meinertzhagen (2010). Based on anatomy and, for some cases, marker gene expression, fenestrated and pseudo‐cartridge glia are believed to be specialized types of PNG and SPG, distal and proximal satellite glia specialized CG, epithelial glia specialized ALG, and marginal glia specialized EG (Fig. 9A.1).

**Figure 8:**
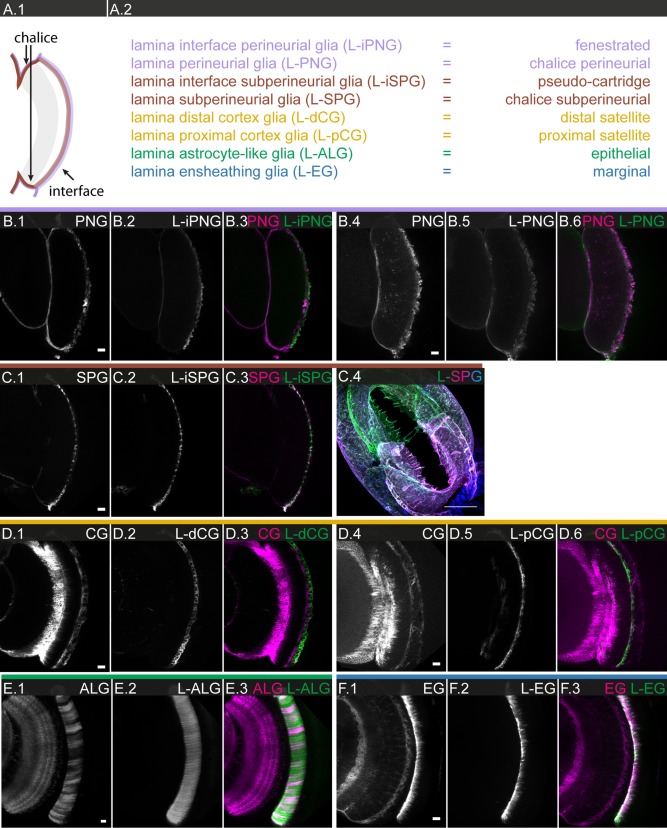
**Equivalency of lamina‐specific glial subtypes with generic glial subtypes**. **A.1** Schematic of lamina and its interfaces. **A.2** Generic and lamina‐specific nomenclature for glial subtypes. **B.1–3** Equivalency of generic perineurial glia (**B.1**) and lamina fenestrated glia (**B.2**), merged in **B.3**; single section. **B.4–6** Equivalency of generic perineurial glia (**B.4**) and lamina‐specific perineurial chalice glia (**B.5**), merged in **B.6**; single section. **C.1–4** Equivalency of generic subperineurial glia (**C.1**) and lamina‐specific pseudo‐cartridge glia (**C.2**), merged in **C.3**; single section. **C.4** Structure of subperineurial glia chalice; 84 µm projections. **D.1–3** Equivalency of generic cortex glia (**D.1**) and lamina‐specific distal satellite glia (**D.2**), merged in **D.3**; single section. **D.4–6** Equivalency of generic cortex glia (**D.4**) and lamina‐specific proximal satellite glia (**D.5**), merged in **D.6**; single section. **E.1–3** Equivalency of generic astrocyte‐like glia (**E.1**) and lamina‐specific epithelial glia (**E.2**), merged in **E.3**; single section. **F.1–3** Equivalency of generic ensheathing glia (**F.1**) and lamina‐specific marginal glia (**F.2**), merged in **F.3**; single section. Scale bar = 10 µm in all images.

In the Janelia GAL4 collection, we identified driver lines specifically expressed in each of these lamina glial subtypes (Supp. Info. Table S2). We were able to establish equivalency with the generic glial subtypes by combining lamina glial drivers with generic glial drivers and appropriate reporters using the orthogonal expression systems GAL4:UAS and LexA:LexAop and checking for coexpression of the two drivers in the same cell populations (Fig. 8). Given the extremely stereotyped and reiterative neuronal organization of the lamina, we are particularly interested in determining whether the glial structures are in register with the neuronal structures, and whether the different glial subtypes behave in the same or different fashion.

#### Lamina surface and cortex

The two distal‐most layers of the lamina are thin and made up by the fenestrated and pseudo‐cartridge glia, which enwrap the entrance points of the photoreceptor axon bundles (Edwards et al., 2012). While topologically equivalent to the PNG and SPGl layer, these cells at the retina‐lamina interface show morphological specializations not found elsewhere on the surface of the brain. Due to technical limitations, we were only able to recover pseudo‐cartridge glia in our preparations.

**Figure 9:**
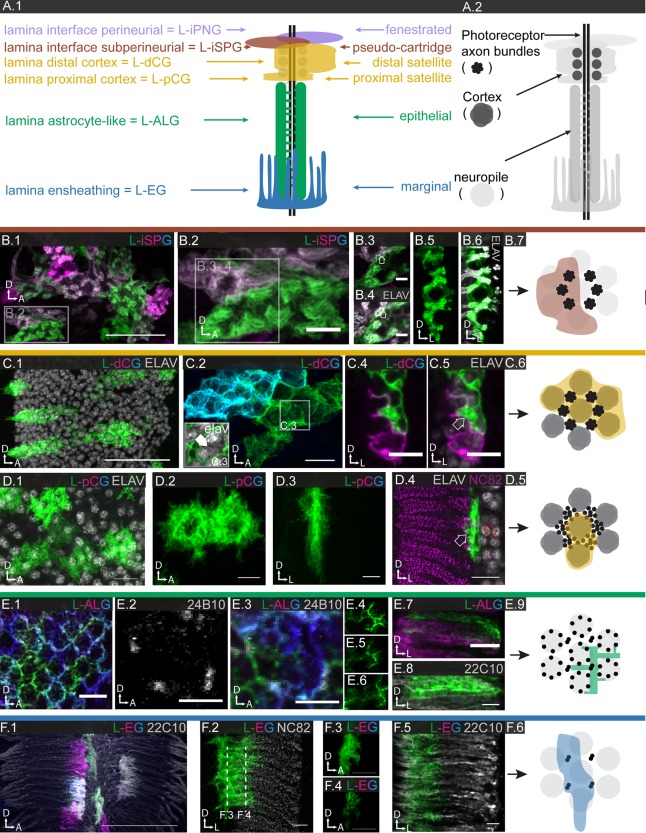
Glial cell morphologies and glia‐neuron interactions in the lamina. **A.1,2**: Schematics illustrating the glial **(A.1)** and neuronal **(A.2)** components of a lamina column. All generic glial subtypes are present but show lamina‐specific morphologies, inspiring the distinct nomenclature for lamina glia. **B.1–6**: Individual cell morphologies of L‐SPG; **B.1,2**: 5 µm projection; **B.3,4**: single sections; **B.5,6**: 3 µm projections. **B.1,2**: L‐SPG are large oblong cells extended along the a‐p axis, with each cell enveloping multiple incoming retinula fibers. **B.3.6** Fine protrusions of the L‐SPG ensheath incoming retinula fibers on the distal lamina surface (arrows in **B.3,4**). **B.7**: Schematic of relationship between L‐SPG and retinula fibers. **C.1–5**: Individual cell morphologies of lamina distal cortex glia (L‐dCG); **C.1**: 42 µm projections; **C.2**: 6 µm projection; **C.4,5**: single sections. **C.1**: L‐dCG are uniformly large and oblong in shape along the a‐p axis, with each cell enveloping multiple cartridges. **C.2**: L‐dCG form honey comb‐like structures that house lamina neuron cell bodies. Note that two L‐dCG may jointly form one honey comb. **C.3**: L‐dCG ensheath neuronal cell bodies as well as tracts. More than one neuronal cell body can be found within one L‐dCG pocket (arrow). **C.4,5**: L‐dCG cells partially lie on top of each other (arrow in **C.5**). **C.6** Schematic of relationship between L‐dCG, retinula fibers, and lamina neuron cell bodies. **D.1–5**: Individual cell morphologies of lamina proximal cortex glia (L‐pCG); **D.1**: 4 µm projection; **D.2**: 6 µm projections; **D.3**: 12.5 µm projection; **D.4**: single section. **D.1**: L‐pCG are smaller and show more variable orientation along the a‐p axis, as compared with their distal counterparts. **D.2,3**: Higher magnification shows that L‐pCG are thin and compact/dense cells. **D.4**: L‐pCG lie at the interface between lamina cortex and neuropile. On their distal surface, they form pockets (red arrow) for neuronal cell bodies, on their proximal surface, and they send protrusions into the neuropile (white arrow). **D.5**: Schematic of relationship between L‐pCG, retinula fibers and lamina neuron cell bodies. **E.1–8**: Individual cell morphologies of lamina L‐ALG; **E.1–7**: single sections **E.8**: 4 µm projection. **E.1**: L‐ALG form a thin lattice. **E.2,3**: Single lamina cartridge, showing that photoreceptor projections (**E.2**) are in close contact with L‐ALG lattice (**E.3**). **E.4–6**: Distal to proximal sections through single L‐ALG, showing that the main branches of the glial cell are maintained but smaller processes appear and disappear. **E.7,8**: L‐ALG mosaic revealing a mesh‐like structure extending through the lamina neuropile. **E.9**: Schematic of relationship between single L‐ALG and lamina columns. **F.1–5**: Individual cell morphologies of lamina ensheathing glia (L‐EG); **F.1**: 30 µm projection; **F.2**: 3 µm projection; **F.3**: 17 µm projection, **F.4,5**: single sections. **F.1**: L‐EG are narrow cells oriented along the dorso‐ventral axis, ensheathing multiple lamina cartridges. **F.2**,**5**: L‐EG send many fine protrusions deep into the lamina neuropile as well as shorter processes medially into the outer chiasm. **F.3,4**: Cross‐sections of single marginal cell at its base (**F.3**) and tip (**F.4**). **F.11**: Schematic of relationship between single L‐EG and lamina columns. Scale bar = 10 µm in **B.1,2**, **C.1,2**, **D.1**, **E.1**, **F.1**; 5 µm in **B.3–5**, **D.2–5**, **E.2,3,7,8**, **F.2–5**.

We counted a total number of ∼100 pseudo‐cartridge glia (R50A12; Supp. Info. Table S2), which are large irregularly shaped cells that extend along the antero‐posterior axis of the lamina (Fig. 9B.1). Colabeling of axons reveals that multiple (6‐10) ommatidial axon bundles are enveloped by a single glial cell (Fig. 9B.1–2). High magnification views show that pseudo‐cartridge glia ensheath individual bundles (Fig. 9B.3–4) but, interestingly, individual glia do not follow the axon tracts along their entire route along the distal‐proximal axis (Fig. 9B.5–6). The observed morphological specialization of the two surface glial subtypes at the retina‐lamina interphase likely reflects the need for maintaining insulation of the CNS while accommodating the massive incoming parallel photoreceptor projections from the retina. Notably, the outer perimeter of the lamina whose surface forms a chalice‐like structure is also covered by PNG and SPG (Fig. 8B.4–6, C.4). This chalice connects the retina‐lamina interface with the surrounding brain surface.

Directly beneath the surface lies the lamina cortex which harbors the cell bodies of the lamina neurons and two layers of glial cells (Edwards et al., 2012), the distal (R53B07) and proximal (R44B12) satellite glia. While topologically equivalent to CG (R77A03; Fig. 8D.1–6), we did not find two morphologically distinct layers of glia in any other cortex region of the brain.

We counted a total of ∼125 distal satellite and ∼250 proximal satellite glia (Supp. Info. Table S2). The distal satellite glia are uniformly large and oblong in shape, extending along the anterior‐posterior axis of the lamina (Fig. 9C.1). They form a thin honeycomb‐like structure that houses the lamina neuron cell bodies and ensheath the neuronal tracts running through the distal part of the cortex (Fig. 9C.2). Unlike elsewhere, more than one neuronal cell body can be found within one glial pocket (Fig. 9C.3). However, as elsewhere, neighboring distal satellite glia frequently collaborate in enclosing neuronal entities, along all three axes: Within the plane of the cortical layer, two glial cells may contribute to forming the honeycomb that encloses a single lamina column (Fig. 9C.2). Along the proximal‐distal axis, neighboring distal satellite glial cells overlap and secure the enclosure of the cartridge along its long axis (Fig. 9C.4–5).

By comparison, the proximal satellite glia have very distinct morphological characteristics. The cells are smaller and show more variable orientation along the anterior‐posterior axis as compared to their distal counterparts (Fig. 9D.1). The cells are compact and form a dense layer at the interface of lamina cortex and neuropile (Fig. 9D.2–3). On their distal surface, they form pocket‐like structures that cap neuronal cell bodies, on their proximal surface, they send protrusions into the synaptic region (Fig. 9D.3–4).

#### Lamina neuropile

The next proximate layer is deep and comprises the neuropile portion and the epithelial glia (R55B03) of the lamina (Edwards et al., 2012; Meinertzhagen and O'Neil, 1991), which we show to be a specialized form of ALG (Fig. 8E.1–3). We counted a total number of ∼470 epithelial glia (Supp. Info. Table S2). They form a well‐ordered lattice in which the synaptic portions of the lamina columns are ensconced (Fig. 9E.1). Interestingly, individual lamina columns are not wrapped by one glial cell; instead, epithelial glia are located at the corners and thus contribute to the wrapping of multiple columns (Fig. 9E.2–6). The main branching/segmentation pattern of an individual epithelial glia is maintained along the distal‐proximal axis, however, the glia also form smaller protrusions into the column, which appear to be more localized (Fig. 9E.4–6). Incoming photoreceptor axons are found in close association with the epithelial glial lattice. The mesh‐like micro‐morphology, a hallmark of ALG, is best visible in horizontal views (Fig. 9E.7–8).

Finally, the marginal glia (R60F04), which we show to be a specialized form of EG (Fig. 8F.1–3), form a contiguous layer at the proximal margin of the lamina (Edwards et al., 2012). We count ∼100 marginal glia (Supp. Info. Table S2). They are long, narrow cells oriented along the dorso‐ventral axis of the lamina and associate with multiple lamina columns (Fig. 9F.1). Colabeling with a synaptic marker shows that marginal glia ensheath the proximal portion of the cartridges and extend processes of variable length into the bottom third of the neuropil layer (Fig. 9F.2–5). Marginal glia also send protrusions proximally into the outer chiasm (Fig. 9F.2).

Our analysis of the lamina glia yields several interesting insights. Despite significant morphological specialization of the lamina glia, they can all be subsumed under the generic glial subtypes based on marker gene expression. In contrast to the highly stereotyped number of neurons and projections, and their well‐defined organization into lamina columns, each glial subtype is present in different numbers, shape, and orientation. Both globally and locally, the glial structures register neither with the neuronal structures nor with each other. As described for the glial subtypes of other brain regions, lamina glia show global tiling, homotypic and heterotypic filopodial contact, task sharing through collaboration with neighbors and relay with other glial subtypes.

### The Medulla

**Figure 10:**
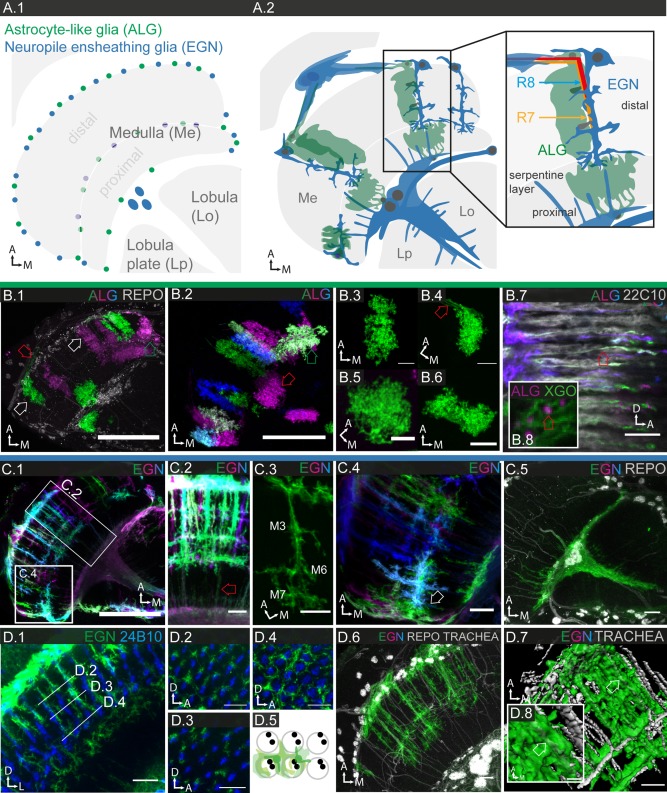
Glial cell morphologies in the medulla and interactions with neurons. **A.1**: Schematic showing the location of ALG and EGN cell bodies. Cell bodies are located at the distal and proximal border of the medulla, as well as the serpentine layer, which forms the border between distal and proximal medulla. **A.2**: Schematic depicting the main features of ALG and EGN. A medulla column is magnified and illustrates the relationship between photoreceptor projections and different glial subtypes. **B.1–7**: Individual cell morphologies of ALG; **B.1**: 3 µm projection; **B.2**: 30 µm projection; **B.3–6**: 9–18 µm projections; **B.7,8**: single sections. **B.1–6**: ALG with cell bodies in the distal medulla cortex occupy the distal medulla, forming irregular columnar structures (white arrows in **B.1**) and sending fine processes toward the outer chiasm (red arrows in **B.1**,**4**). ALG with cell bodies in the proximal medulla cortex occupy the proximal medulla, forming irregular square‐like structures (“chandelier glia,” red arrow **B.2**). ALG with cell bodies around the serpentine layer branch out into both distal and proximal medulla (green arrows **B.1,2**). **B.7,8**: ALG send long processes into the outer chiasm (red open arrows). **B.8**. These ALG processes lie within the sheath of chiasm glia next to neuronal tracts. **C.1–5**: Individual EGN cell morphologies**; C.1–3**: 3–5 µm projections; **C.4**: 8 µm projection; **C.5**: 11 µm projection. The EGN in the distal medulla are organized in highly columnar structures (**C.1**,**2**) and show a characteristic branching pattern in layers M3, M6, and M7. The fine processes in the proximal medulla (red open arrow in **C.2**) belong to the inner chiasm glia. The EGN in the serpentine layer send columnar branches into both distal and proximal medulla (white arrow **C.4**). The EGN of the inner chiasm send fine protrusions into the proximal medulla (**C.5**). **D.1–5**: EGN accompany photoreceptor projections, which terminate in medulla layers M3 and M6. **D.1**: 3 µm projection; **D.2–4**: Cross sections (3 µm projections) through M3 (**D.2**), in between (**D.3**) and M6 (**D.4**), showing differences in the density of the glial ensheathment. **D.5**: Schematic illustrating the association of individual EGN with medulla columns. **D.6–8**: EGN partially envelop (arrows) the many tracheal branches that invade the medulla. **D.7,8**: 3D reconstruction of a substack of **D.6**. Scale bar = 50 µm in **B.1,2**, **C.1**; 5 µm in **B.3–8**, **C.2–5**, **D.1–4**, **D.6,7**; 2 µm in **D.8**.

The medulla is the second optic relay station onto which photoreceptors R7‐R8 and all lamina interneurons project in a retinotopic fashion. However, compared to the lamina, horizontal connections become more prominent in the medulla, leading to the co‐existence of layered and columnar structures. Altogether, the medulla neuropile comprises ten distinct layers of synaptic connections (M1‐M10) that are organized into retinotopic columns (Fischbach, 1989; Morante and Desplan, 2008). Photoreceptor R8 terminates in layer M3, photoreceptor R7 in layer M6. Notably, the medulla derives from two distinct primordia; only during pupariation, distal (M1‐M6) and proximal (M7‐M10) medulla fuse at the serpentine layer (M7; Fig. 10A.1; Hofbauer and Campos‐Ortega, 1990; Meinertzhagen, 1993).

In the Janelia GAL4 collection, we identified lines with medulla‐specific expression, but in all cases, the expression was somewhat mosaic. The medulla cortex contains CG that are morphologically similar to those found in the central brain (Awasaki et al., 2008). The neuropile is populated by astrocyte‐like and EG (Edwards et al., 2012; Hadjieconomou et al., 2011). The cell bodies of both types of glia are found surrounding the medulla neuropile, but also in the serpentine layer, which forms the boundary between distal and proximal medulla. Using these medulla‐specific drivers (R31E10, R73B10), we count ∼350 and ∼370 cells (Supp. Info. Table S2), respectively, which is an underestimate but nevertheless suggests roughly a 1:1 ratio between astrocyte‐like and EG, the same ratio observed elsewhere in the brain.

For ALG, we find three types of cellular arrangements, depending on the location of the glial cell body (Fig. 10B.1–6). ALG with cell bodies located in the distal medulla cortex occupy the distal medulla, forming irregular columnar structures and sending fine long processes into the outer chiasm (Fig. 10B.1 and B.7). These processes run alongside neuronal tracts and are ensheathed by outer chiasm glia (Fig. 10B.7–8). ALG, whose cell bodies are located in the proximal medulla cortex, occupy the proximal medulla and form irregular square‐shaped structures (Fig. 10B.2, 5). Finally, ALG, whose cell bodies are located in the cortical circumference of the serpentine layer, branch out into both distal and proximal medulla (Fig. 10B.2, 6). All ALG in the medulla show tiling and manifest a high structural density, which is associated with low synaptic density.

For EG, we also find three types of situations (Fig. 10C). EG, whose cell bodies are located in the distal medulla cortex, invade the distal medulla as highly columnar structures (Fig. 10C.1–2). These glia show a characteristic branching pattern around layers M3 and M6, where photoreceptors R8 and R7 terminate, and in M7, the serpentine layer (Fig. 10C.3). Both at the distal margin and in the serpentine layer, these EG form a rather dense sheath. EG with cell bodies located in the cortical circumference of the serpentine layer send columnar branches into both distal and proximal medulla (Fig. 10C.4). However, we find no cell bodies of EG at the proximal margin of the medulla (Fig. 10A.1). Instead, we find that the proximal medulla is invaded by many fine processes from the inner chiasm glia (Fig. 10C.5).

To have a closer look at the distal EG, we colabeled the incoming photoreceptor axons. In all layers, the photoreceptor axons are closely associated with the EG, however, in the layers in which photoreceptor axons terminate the glial processes are enlarged (M3) or even form a broad meshwork (M6; Fig. 10D.1–5). Finally, we examined the relationship between EG and the tracheal network, which pervades the medulla mostly in a columnar fashion. We observe close association of glia with tracheal branches and 3D reconstruction suggests that tracheal branches are largely if not completely covered by the EG (Fig. 10D.6–7; Pereanu et al., 2007). Overall, the columnar organization of the medulla neuropile is reflected in the columnar shape of both astrocyte‐like and EG, its layered structure in the branching pattern of the EG.

### The Lobula Complex

**Figure 11:**
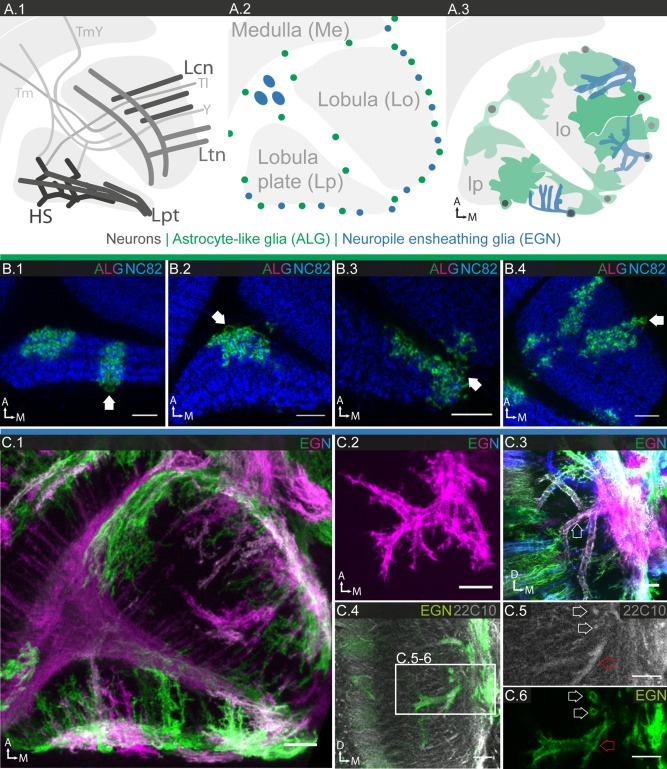
Glial cell morphologies in the lobula complex. **A.1–3**: Schematics of lobula complex, consisting of lobula and lobula plate, and neighboring medulla. **A.1**: Main neuronal connections; transneurons connect medulla and lobula complex. Lobula and lobula plate contain large dendritic arborizations of visual PNs. **A.2**: Location of ALG and EGN cell bodies. The cell bodies of large inner chiasm glia (XGI) are located between proximal medulla and lobula complex. **A.3**: Main morphologic features of ALG and EGN. **B.1–4**: Individual ALG cell morphologies; 1 µm sections. In both lobula and lobula plate, ALG take on various shapes and sizes and can send branches into both lobula and lobula plate; location of cell bodies marked by white arrows (**B.1–4**). ALG entering the lobula from the anterior margin show a (loosely) columnar organization (**B.4**). **C.1–6** Individual EGN cell morphologies; **C.1**: 12.5 µm projection; **C.2**: 10 µm projection; **C.3**; 10 µm projection; **C.4–6** single sections. **C.1** EGN have column‐like processes perpendicular to the margin, as well as tangential processes parallel to the margin. **C.2,3**: EGN form a complex three‐dimensional network of branches. **C.4–6** EGN enclose the large dendritic arborizations of lobula plate tangential cells. **C.6** Cross sections of single tubes reveal that neuronal processes are completely enclosed (white and red arrows). Scale bar = 10 µm in all images.

The lobula complex comprises the lobula plate, which is involved in motion processing (Borst et al., 2010), and the lobula, which is involved in higher order color, motion processing and small object motion detection (Morante and Desplan, 2008; Zhang et al., 2013). Transneurons connect medulla to lobula and/or lobula plate, and lobula to lobula plate. Both lobula and lobula plate contain large dendritic arborizations of visual projection neurons (PN; Fig. 11A.1; Fischbach, 1989; Hausen et al., 1980; Otsuna and Ito, 2006; Scott et al., 2002). Compared with lamina and medulla, horizontal connectivity prevails in the lobula complex neuropiles. The glia of the lobula complex have been examined (Edwards and Meinertzhagen, 2010; Tix et al., 1997).

ALG enter the lobula and lobula plate predominantly from the outer margins (medial/anterior margin of lobula plate, lateral/anterior margin of the lobula) and only rarely from the inner chiasm (Fig. 11A.2). ALG in the lobula complex show a much greater variation in shape than those of lamina and medulla (Fig. 11B.1–4), however, the glia that invade the lobula from the anterior margin show a more columnar organization (Fig. 11B.4). Not infrequently, the glia form two branches, which may invade two of the three neighboring neuropiles (lobula/lobula plate/medulla; Fig. 11B.3). Overall, the glial cells show a high structural density, corresponding to the relatively lower synaptic density in the lobula complex (see also Fig. 5B.3–4).

EG send large projections from the outer margins into lobula and lobula plate, while inner chiasm glia send fine protrusions from the inner margins into all three neighboring neuropiles (medulla, lobula, lobula plate) (Fig. 11C.1). In both lobula and lobula plate, EG form columnar processes perpendicular to the neuropile margin, as well as tangential processes parallel to the margin (Fig. 11C.1). Higher magnifications reveal complex three‐dimensional networks of branches (Fig. 11C.2–3); colabeling shows that neuronal processes appear to be completely enclosed (Fig. 11C.4–6).

### The Chiasms

**Figure 12:**
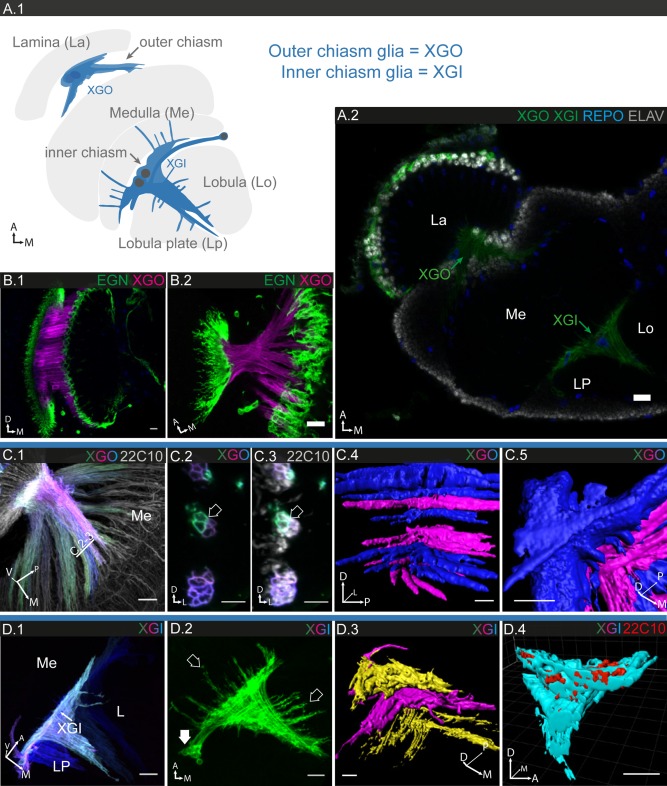
The glia of the outer and inner chiasm. **A.1**: Schematic of the optic lobes and their chiasms. The outer chiasm connects lamina and distal medulla, the inner chiasm connects proximal medulla, lobula and lobula plate. **A.2**: Labeling of chiasm glia (XG) in outer (XGO) and inner (XGI) chiasm; 1 µm section. **B.1,2**: XG and EGN are distinct populations in outer chiasm; **B.1**: 15 µm projection; **B.2**: 4 µm projection. **C.1–5**: Individual XG cell morphologies in outer chiasm; **C.1**: 30 µm projection; **C: 2,3**: single sections. Neuronal tracts are enveloped by XGO (arrows in **C.2,3**). **C.4,5**: 3D reconstruction of **C.1**, view from medial medulla into chiasm (**C.4**), view onto lamina portion of chiasm (**C.5)**. Different fibers crossing the chiasm at perpendicular angles can be ensheathed by a single XGO cell. **D.1–4** Individual XG cell morphologies in inner chiasm; **D.1**: 93 µm projection; **D.2**: 60 µm projection. **D.1**: A single XGI cell may extend its sheath‐like processes along the entire coronal plane of the inner chiasm. **D.2**: XGI send many long protrusions (open arrows) into all three neighboring neuropiles. Most XGI cell bodies lie outside the chiasm (filled arrow). **D.3,4**: 3D reconstruction of **D.1**, view of the dorso‐ventral axis showing the multilayered structure of the chiasm (**D.3**); **D.4**: cross section through **D.3,** XGI envelop neuronal tracts. Scale bar = 10 µm in all images.

The optic lobes contain two major tracts that connect the different neuropiles, which are different with regard to function and morphology. The outer chiasm connects lamina and medulla. During larval and early pupal stages, retinotopic projections are made by the R7‐R8 axons and lamina interneurons from the lamina into the medulla. The crossover of fibers (‘chiasm’) occurs in the pupa, after the lamina‐medulla connections are established, due to a 90° rotation of the lamina against the medulla neuropile (Braitenberg, 1970; Meinertzhagen, 1993). The inner chiasm connects the medulla with the lobula complex; fibers connecting medulla with lobula and lobula plate, as well as lobula with lobula plate, run through this chiasm, leading to a massive criss‐crossing of fibers (Fig. 12A‐1; Braitenberg, 1970; Meinertzhagen, 1993). Glial cells are present in both chiasms (Tix et al., 1997).

In the Janelia GAL4 collection, we identified only very few drivers that are specifically expressed in chiasm glia. In most cases, expression in chiasm glia is mixed with expression in EG. Interestingly, expression in the inner chiasm glia is always associated with expression in EGN, while expression in the outer chiasm glia is frequently associated with expression in EGT, suggesting an underlying difference between the glia populating the two chiasms. Only one of the drivers (R53H12) is expressed in both outer and inner chiasm (Fig. 12A.2). Our cell count places the number of outer chiasm glia at ∼50, suggesting that, on average, one glial cell enwraps 15 lamina‐medulla fiber tracts; the number of inner chiasm glia is ∼40 (Supp. Info. Table S2). The nuclei of the outer and inner chiasm are large and located in the middle of the chiasm regions (Tix et al., 1997). The outer chiasm glia is clearly distinct from the glia ensheathing the proximal lamina and the distal medulla neuropiles (Fig. 12B.1–2); unlike inner chiasm glia (see below), outer chiasm glia do not invade the neuropiles they connect. MCFO and co‐labeling of axon tracts reveals that lamina‐medulla connections are bundled into larger strands (Fig. 12C.1). A single outer chiasm glia cell envelopes several (8‐12) fiber bundles (Fig. 12C.2–3) emerging from neighboring lamina cartridges and accompany them to the medulla surface, where the fiber bundles fan out again (Fig. 12C.1, C.5). However, more than one glial cell participates in wrapping fibers within any large strand; in cross section, multiple enclosures are visible in which small sets of neuronal tracts run (Fig. 12C.2–3). The absence of exclusivity is in keeping with the generally observed lack of registration between glial and neuronal entities. The inner chiasm glia are elongated sheet‐like cells (Fig. 12D.1), which send long, fine protrusions into the adjacent neuropiles (Fig. 12D.2). Collectively, they form triangular structures that are stacked on top of each other (Fig. 12D.2–3) and connect the three neuropiles (Fig. 12D.2–3) by enveloping all neuronal tracts running through the inner chiasm (Fig. 12D.4).

### Glia in the Olfactory System

Finally, we examined glia‐neuron interactions in the olfactory system. Similar to the visual system, the olfactory system makes up a significant portion of the fly brain and has been investigated in great detail anatomically and functionally (for review see Imai et al., 2010; Komiyama and Luo, 2006; Masse et al., 2009; Su et al., 2009; Vosshall and Stocker, 2007; Wilson, 2013), including several studies on its glia (Doherty et al., 2009; Ito et al., 1997; Leiss et al., 2009; Oland et al., 2008; Sen et al., 2005; Sinakevitch et al., 2010). Like the visual system, the olfactory system shows a topographical organization and comprises several relay stations, from the antennal lobe to the higher olfactory centers mushroom body and lateral horn. Importantly, the neuronal architecture is well understood and excellent neuronal GAL4 drivers are available, which in combination with our newly generated glial LexA drivers allowed us to examine the interactions between glia and neurons in exquisite detail in the different regions of the olfactory system, in particular the pre‐ and postsynaptic compartments.

### The Antennal Lobe

**Figure 13:**
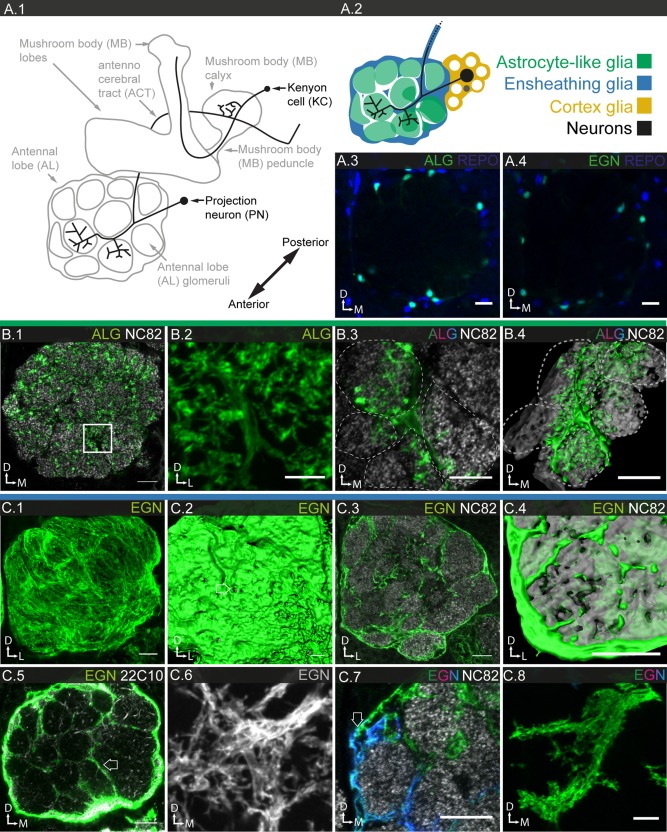
Characterization of glia in the antennal lobe. **A.1**: Schematic of the olfactory system and its major neuronal connections by PNs that connect the antennal lobe with higher brain centers and by mushroom body intrinsic KC. **A.2**: Schematic of antennal lobe and its glial subtypes, ALG and EGN. **A.3,4**: Location of glial cell bodies; single sections. ALG (**A.3**) and EGN (**A.4**) cell bodies are found surrounding the antennal lobe neuropile but not inside. **B.1–4**: ALG in the antennal lobe. **B.1,2**: General expression pattern; **B.1**: single section; **B.2**: 2 µm projection. ALG show a low density of processes throughout the antennal lobe (**B.1**), high magnification reveals thick branches, fine processes, and occasional thickenings. **B 3,4**: Single ALG cell morphology; **B.3** single section; **B.4**: 3D reconstruction of cell in **B.3**. Individual ALG cells penetrate multiple glomeruli with their processes without any anatomical registration. **C.1–8**: EGN in the antennal lobe. **C.1–6**: General expression pattern; **C.1**,**6**: 3.5 µm projection; **C.3,5**: single sections. **C.1**: EGN processes cover the surface of the antennal lobe. **C.2**: 3D reconstruction of **C.1**: showing that the glial sheath is nearly contiguous, with small holes remaining for nerve entries (white arrow). **C.3**: EGN processes projecting between the glomeruli. **C.4**: 3D reconstruction of **C.3** showing that, in contrast to the antennal sheath, the glial cover of individual glomeruli is not continuous. **C.5**: Neuronal tracts enter the antennal lobe along the glomerular boundaries and are in contact with glial processes (white arrow). **C.6**: High magnification of EGN at glomerular boundaries reveals tube‐like morphologies. **C.7,8**: Single EGN cell morphologies; **C.7**: single section; **C.8**: 12.5 µm projection. **C.7**: Neighboring EGN cells cover distinct areas but partially interdigitate in regions of contact (white arrow). **C.8**: Individual EGN are not confined to single glomeruli. From the main glial processes along the glomerular boundaries smaller branches project perpendicularly into different glomeruli. Scale bar = 10 µm in **A.3**,**4**, **B.1**,**3**, and **C.1–5**; 5 µm in **B.2**, **C.7,8**.

Approximately 1,300 olfactory sensory neurons (OSNs) project from the antenna and maxillary palp onto ∼50 distinct glomeruli in the antennal lobe, which contain local interneurons (LNs) and ∼150 PNs. The OSNs that express the same odorant receptor (OR) project to one spatially invariant glomerulus within the antennal lobe (Couto et al., 2005; Fishilevich and Vosshall, 2005), each populated by a distinct class of PNs. The PNs send their dendrites into the glomeruli of the antennal lobe and project their axons to the higher olfactory centers (Fig. 13A.1).

The cortex of the antennal lobe contains CG, the neuropile is populated by both astrocyte‐like and EG (Fig. 13A.2). However, in contrast to the visual system, we could not identify antennal lobe‐specific drivers for any of the three glial subtypes in the Janelia GAL4 collection, and thus based our analysis on generic drivers and multicolor mosaic experiments.

Similar to other regions in the brain, the cell bodies of all three glial subtypes are found surrounding the surface of the antennal lobe but not within the neuropile (Fig. 13A.3–4, iv). CG surround the entire antennal lobe and enclose the neuronal cell bodies of (LNs and) PNs (see Fig. 15B.1–3). ALG show an evenly high density of extensions throughout the entire neuropile, without any significant differences between individual glomeruli (Fig. 13B.1). Individual ALG are not confined to a single glomerulus (Fig. 13B.3–4) but rather spread across multiple glomeruli. High magnification views of individual ALG reveal a highly ramified morphology, studded with locally increased densities (Fig. 13B.2).

EG cover the entire neuropile of the antennal lobe with a very thin sheet (Fig. 13C.1); the small holes visible in the sheet may provide entry for incoming neuronal projections (Fig. 13C.2). Moreover, the EG send extensions in between the glomeruli (Fig. 13C.3). 3D reconstructions show that while the antennal lobe is covered by a nearly contiguous sheet, the individual glomeruli are not (Fig. 13C.4; cf (Oland et al., 2008)). Many of the EG extensions into the glomeruli appear as tube‐like structures (Fig. 13C.6, 8), and double‐labeling showed that these glial extensions strongly colocalize with dendritic/axonal processes (Fig. 13C.5), reminiscent of the micromorphology seen in EG in the lobula complex of the visual system. MCFO experiments reveal the glia as large, elongated cells of irregular shape. The cells do not physically overlap but frequently interweave with one another. Optical cross‐sections through the lobe show that the extensions of individual EG reach deep into the neuropile, often contacting several glomeruli (Fig. 13C.7). The cells have a lamellar (velate) morphology, with fine processes branching out into the glomeruli themselves (Fig. 13C.8).

### The Mushroom Body

**Figure 14:**
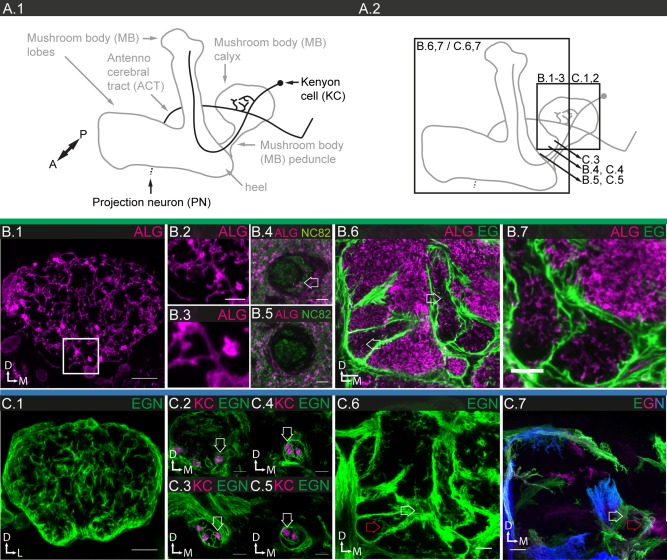
Characterization of glia in the mushroom body. **A.1**: Schematic of the olfactory projections in the mushroom body. **A.2**: Schematic of the mushroom body neuropile and the focal planes represented in the panels below. **B.1–7**: ALG. **B.1–3**,6,7: 5 µm projections; **B.4,5**: single sections. **B.1–3**: General expression pattern revealing low structural density of ALG projections in the calyx. **B.2,3**: Higher magnification showing that ALG processes are highly branched and have occasional thickenings. **B.4,5**: In the peduncle, ALG processes are rare even in the proximity of synapses (**B.4**), but emerge in the transition from peduncle to heel (**B.5**). **B.6,7**: In the mushroom body lobes, the structural density of ALG is low. **C.1–7**: EGN in the mushroom body. **C.1–6**: General expression pattern; **C.1**: 5 μm projection; **C.2–5**: single sections; **C.6**: 10 μm projection. **C.1**: EGN processes permeate the calyx. **C.2–5**: Cross‐sections through different portions of calyx and peduncle. EGN subcompartmentalize the calyx by enwrapping individual subpopulations of KC (white arrows). **C.6**: β, β′, and γ lobes are separated by sheaths of EGN (white arrow); even within the individual lobes, EGN provide further subcompartmentalization (red arrow). **C.7**: Individual EGN cell morphologies in the lobes; 10 μm projection. Both sheath‐like structures and fine protrusions are visible (white and red arrow). Scale bar = 10 µm in **B.1–3**, **C.1–7**, **D.1**,**6,7**, **E.1–7**; 5 µm in **D.2–5**.

The higher order centers of the olfactory system, lateral horn and mushroom body, are innervated by the PNs via antenno‐cerebral tracts. En route to the lateral horn, the 200 PNs send collaterals into the mushroom body, the center of olfactory learning. The mushroom body is composed of some 2,500 Kenyon cells (KC). In the calyx, each PN makes divergent, moderately stereotyped, synaptic connections with 2–11 KC; in turn, the KC send axonal projections through the peduncle into different lobes within the mushroom body and are thereby subdivided into three distinct subsets (α/β, α’/β’, γ; Fig. 14A.1; for review: Davis, 2011; Fiala, 2007; Jefferis et al., 2007; Lin et al., 2007; Murthy et al., 2008; Tanaka et al., 2004).

As in other regions of the brain, the cortex of the mushroom body contains CG, the neuropile regions are populated by both astrocyte‐like and EG. As in the case of the antennal lobe, we could not identify mushroom body‐specific drivers for any of the three glial subtypes within the Janelia GAL4 collection, and thus base our analysis on generic glia drivers, in particular glia‐LexA/neuron‐GAL4 colabeling, and multicolor mosaic experiments.

As elsewhere, the presence of ALG coincides with the presence of synapses, and the density of glial processes is anticorrelated with the density of synapses. The calyx and the lobes of the mushroom bodies are particularly synapse‐rich neuropile regions and pervaded by a low density of astrocyte‐like processes (Fig. 14B.1,6‐7). The glial processes have an uncommon morphology; overall, they show thin branches with local thickenings at various positions within their network (Fig. 14B.2–3). The peduncle, a tract region, is sparse in synapses and contains very few astrocyte‐like processes (Fig. 14B.4–5).

EG envelop the outer bounds of the calyx but, surprisingly, show a vast number of extensions that permeate its inner structure (Fig. 14C.1). Colabeling of a subset of KC reveal that their projections are enwrapped by EG and that, thereby, different neuronal subpopulations are kept sequestrated from the calyx onwards throughout the peduncle (Fig. 14C.2–5). EG envelop the mushroom body lobes and permeate their inner structure with numerous extensions as well (Fig. 14C.6). MCFO experiments confirm these two morphological features, sheath formation as well as protrusions that enwrap neuronal processes (Fig. 14C.7).

### Neuron‐Glia Interactions along the Projections of the Olfactory Projection Neurons

**Figure 15:**
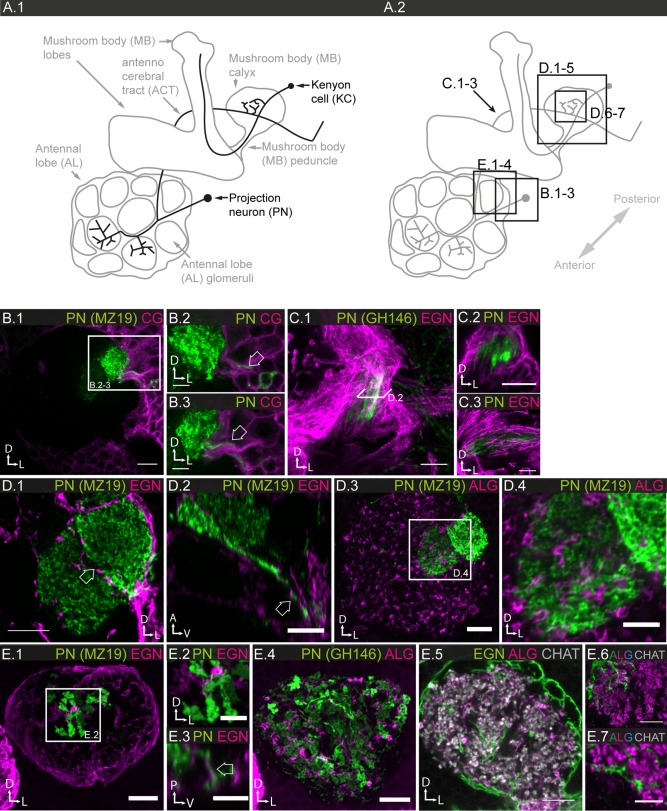
Characterization of the glia‐neuron interactions in the olfactory system. **A.1,2**: Schematic of the olfactory system and its major neuronal projections. The focal planes of the different panels are shown in **A.2**. **B.1–3**: Spatial relationship between the cell bodies of PN and CG in the antennal lobe cortex; **B.1**: 2 µm projection; **B.2,3**: single sections. Both the neuronal cell body and the neuronal processes (arrows) that project from the cell body to the neuropile region are ensheathed by CG extensions. **C.1–3**: PN axons within the antenno‐cerebral tract are ensheathed by EGN; 2.5 µm projections. The tract is completely enclosed. **D.1,2**: EGN at glomerular boundaries in the antennal lobe; **D.1**: 1.5 µm projection; **D.2**: horizontal projection of **D.1**. **D.1**: The glomeruli of the antennal lobe are ensheathed by a discontinuous layer (arrow) of EGN. **D.2**, EGN extensions accompany neural processes until they enter the neuropiles (arrow). **D.3,4**: ALG; **D.3** 2 µm projection; **D.4** Magnification of **D.3**. The bushy postsynaptic compartment of PNs are intertwined with ALG protrusions, but not all postsynaptic regions are directly in contact with the glia. **E.1–7**: Spatial relationships of EGN and ALG with pre‐ (**E.1–3**) and post‐ (**E.4–7**) synaptic compartments in the calyx. **E.1,2**: 2.5 µm projections; **E.3**: horizontal projection of **E.1**. EGN processes contact PN extensions until they diverge into different terminal regions. **E.4**,**6**: single sections; **E:5**: 1.5 µm projection. Both ALG and EGN processes are found in proximity to postsynaptic regions, but do not closely associate with them in a stereotypic manner. **E.7: 7** Association of ALG with PN presynaptic boutons in calyx; 1.5 µm projections. ALG processes loosely intermingle with sites of synaptic connections but lack specific association with the presynaptic compartment. Scale bar = 10 µm in **B.1**, **C.1**, **D.1**,**3**, **E.1**,4; 5 µm in **B.2,3**, **C.2,3**, **D.2**,**4**, **E.2,3**,**5–7**.

We examined the interaction between PNs and the different glial subtypes from the antennal cortex to the mushroom body calyx, using neuron‐GAL4/glia‐LexA colabeling. CG enwrap the cell bodies of PNs and accompany their projections toward the (antennal) neuropile region (Fig. 15B.1–3). EG, which form a discontiguous sheet around the glomeruli of the antennal lobe, take over and accompany neuronal projections into the neuropile (Fig. 15D.1–2). ALG form a sponge‐like structure surrounding the bushy post‐synaptic compartment of PNs; interestingly, not all postsynaptic regions are visibly in direct contact with the glia (Fig. 15D.3–4).

In the inner antenno‐cerebral tract, EG completely enclose the projections of the PNs (Fig. 15C.1–3) and accompany them to higher order olfactory centers. PN projections entering the mushroom body calyx are accompanied by EG until they subdivide into different branches and terminate as PN boutons (Fig. 15E.1–3). ALG processes loosely intermingle with sites of synaptic connections but lack a systematic association with the presynaptic boutons/compartments (Fig. 15E.4). Similarly, both astrocyte‐like and EG are found in the proximity of AChR‐positive pre‐ and post‐synaptic sites (Wilson, 2013) but lack a stereotyped specific association (Fig. 15E.5–7).

## Discussion

The analysis of glia presented here greatly extends previous studies (Awasaki et al., 2008; Doherty et al., 2009; Edwards and Meinertzhagen, 2010; Edwards et al., 2012) of the different glial subtypes in the adult *Drosophila* nervous system. We provide a high‐resolution description of the morphology of the glia themselves as well as their relationship with other glia and neuronal entities, revealing several fundamental rules governing these interactions. The GAL4 drivers we identified that are specifically expressed in particular glial subtypes permitted a precise determination of the cell numbers for the different glial subtypes, and, in conjunction with morphological criteria, helped assign glia to specific subtypes.

### Expression Patterns and Glial Subtype Specificity

We have used the GAL4 lines and their expression patterns as a tool to characterize glial anatomy, but the definition of glial cell types and their subdivision into different subtypes principally relies on the cells’ location, morphology, and neuronal interaction. However, the fact that many lines exist in which expression and cell type specificity are closely correlated, suggests that marker expression can be used to help define different glial subtypes. In two instances, the use of GAL4 drivers as cell‐type specific markers has been particularly informative. First, the lamina shows the most distinct glial morphologies and has the most region‐specific lines of all the brain regions, consistent with the general notion that morphological specialization is reflected in expression pattern. However, by comparing driver patterns, we could establish equivalency of lamina‐specific and generic glial subtypes and thereby underscore their underlying communality. Second, the fact that nearly all lines expressed in EG are expressed in varying subsets of EGN and EGT supports the notion that they indeed belong to the same subtype.

### Size of the Different Populations of Glial Subtypes

The number of neurons in the brain/CNS has been estimated at ∼150,000 (Jenett et al., 2012). Employing an algorithm that is robust against variation in expression level, we have counted the total number of glia in the CNS using the pan‐glial marker REPO as well as our set of subtype‐specific GAL4 drivers providing the first comprehensive assessment of glial numbers. The total number of (REPO‐positive) glia is 15,700 (+/‐10%) in the entire CNS, i.e. 10% of the total number of cells in the brain; a similar neuron‐to‐glia ratio has been reported for the embryonic nervous system (Ito et al., 1995). Within the margin of error, our cell counts for the different subtypes roughly add up to the pan‐glial cell count (13,500 vs. 15,700); the slight underestimate in the subtype‐glial count may be due to the mild mosaicism in some of the drivers. Overall, our two independent cell counts validate each other and suggest that we have identified all existing glial subtypes.

The two surface glia differ vastly in their numbers, with PNG being nearly ten times as abundant as SPG. CG make up 20% of the entire glial cell population, the remaining glia distribute almost evenly between ALG (34%) and EG (27%), a ratio maintained in both global and regional (lamina, medulla) counts. Our cell counts are consistent with several studies of gliogenesis in the postembryonic brain, which show that the different glial subtypes derive from distinct lineages whose proliferation is governed by different molecular pathways. SPG are maintained from embryogenesis onwards and show little if any expansion (Avet‐Rochex et al., 2012; Awasaki et al., 2008). PNG are generated from superficial precursor cells by symmetric cell division which is driven by paracrine FGFR and InR signaling between neighboring PNG cells (Avet‐Rochex et al., 2012; Awasaki et al., 2008). CG are generated from a different pool of precursor cells also by symmetric cell division but in this case driven by paracrine FGFR signaling from both glial and neuronal neighbors, thereby ensuring coordinated growth of glial and neuronal cell populations (Avet‐Rochex et al., 2012). The adult neuropile glia, astrocyte‐like and EG, are derived from a relatively small set of neuro‐glioblasts, the type II lineages, which proliferate during larval stage. A clonal expansion of the neuropile glia contributes greatly to the mature population (Awasaki et al., 2008; Dearborn and Kunes, 2004; Omoto et al., 2015). Based on NPG‐subtype specific markers, the type II lineages contribute to both astrocyte‐like and EG (Omoto et al., 2015). The equal proportion of the two cell types could thus be due to common lineage or, possibly, by a match‐making mechanism driven by neuronal neighbors (Awasaki et al., 2008; Dearborn and Kunes, 2004), as observed for the CG (Avet‐Rochex et al., 2012) and the longitudinal glia of the embryo (Hidalgo et al., 2011). Studies of the eye imaginal disc suggest that glial cell proliferation is ultimately controlled by the integration of multiple signals from different pathways (Franzdottir et al., 2009; Rangarajan et al., 2001; Reddy and Irvine, 2011).

### All Glial Subtypes Show Tiling but also Localized Contact with Glial Neighbors

Our morphological results reveal several features shared by all glia, suggesting common molecular and developmental mechanisms underlying the generation of nervous system architecture. The relationship between glial cells is characterized by global tiling with little overlap. Glial cells generally minimize physical contact with neighbors of both their own subtype and other subtypes, suggesting some form of homotypic and heterotypic repulsion. Tiling is also observed in vertebrate astrocytes (Brill et al., 2011; Bushong et al., 2004; Livet et al., 2007), but has mostly been studied in the context of dendritic arborization. Tiling is observed in embryonic astrocytes, which are instructed by Prospero, a key effector of Notch in astrogenesis (Peco et al., 2016). In a recent study of tiling in larval astrocytes, Stork et al. (2014) have shown that FGFR signaling is responsible for astrocyte infiltration of the neuropile, which is triggered by neuronal expression of FGF ligands. Ablating neighboring astrocytes results in the expansion of astrocyte territory, suggesting that territories are determined by contact dependent growth inhibition or competition for neuropil growth factors such as the FGF ligands. Whether FGF signaling is involved in the tiling of the other glial subtypes remains to be seen. Since FGF signaling plays a pervasive role in glial development, including proliferation, survival, migration and ensheathment (Avet‐Rochex et al., 2012; Condron, 1999; Franzdottir et al., 2009; Gibson et al., 2012; Shishido et al., 1997), an important question to address will be how specificity is encoded in the repeated signaling.

In addition to the observed global tiling, all glial subtypes show specific, localized, contacts with glial neighbors. PNG and SPG create tight epithelial sheets by establishing well‐defined regions of overlap with their immediate neighbors. The other three subtypes all send fine lamellipodial or filopodial processes not only into their local neighborhood but also far into distant surroundings. We suggest that these contact‐forming protrusions have different adhesive properties than most of the glial cell surface. Examples of regionalized differences in adhesive properties along the cell surface have recently been found in the context of axon guidance (Schwabe et al., 2013). This web of contacts may simply provide physical cohesion for the anatomical structure, but it is interesting to speculate that they might also be used to communicate cellular signals between neighbors as well as more distant regions. Coupling of glia by gap junctions is suspected for several glial subtypes but has been demonstrated for SPG, where they are required to translate metabolic signals into synchronized calcium pulses and insulin secretion (Speder and Brand, 2014), for CG in the lamina (Chaturvedi et al., 2014) and most recently in ALG in the larva (MacNamee et al., 2016), where they are required for neurotransmitter recycling.

### Glial Morphologies Adapt to Neuronal Entities

In their interaction with neurons, glia adapt their macroscopic shape to the neuronal entities they envelop. Surface glia form sheets when covering the body of the CNS, and tubes when covering peripheral nerves. Ensheathing and ALG adjust their shapes to the underlying structure of the neuropile and thus form columnar, cubic, spherical, or even tube‐like structures. A striking demonstration of such morphologic adaptations are found in the different neuropiles and chiasmata of the optic lobes. Similarly, the micromorphology of glia also adapts to the local neuronal environment. EG form sheets from which fine tube‐like structures emanate, each ensheathing one or few neuronal fibers. As we have shown systematically, ALG are characterized by a vast pattern of branches/tufts, whose thickness is inversely proportional to the synaptic density of the neuropiles they invade. This feature is induced locally, since different regions of a glial cell can have different densities. The strict negative correlation of astrocyte and synaptic density we observe in *Drosophila* is contrasted by a more variable relationship observed in vertebrates (“tripartite synapse”), where some neuropils, such as the cerebellum, show high coverage of synapses by astrocytes while others, such as the hippocampus, show lower coverage (Araque et al., 2014; Ventura and Harris, 1999). This suggests that, in lieu of increasing astrocytic structural density, some compensatory mechanisms must exist to deal with high synaptic activity, perhaps by changing their morphology in response to neuronal activity (MacNamee et al., 2016).

All these characteristics suggest that the glia maximize their physical contact with neuronal surfaces. Notably, however, the glia show a complete lack of registration with the neuronal entities they envelop, which is most obvious in structures like the antennal lobe and the lamina. The highly stereotyped neuronal organization of these neuropiles readily reveals that the number of neuronal entities enveloped by the glia is variable and that not only neighboring cells of the same glial subtype, but occasionally those of different subtypes can collaborate in creating full enclosure. This suggests that glia are “attracted” to neuronal surfaces and capable of “burden sharing.” Overall, glia and neurons exist in a many‐to‐many relationship.

All these features of glial behavior—global and local adaptation to shape and size of neuronal entities without sharply defined cell ratios or registration—are consistent with the idea that the final steps of neuronal morphogenesis precede final glial morphogenesis. From fate‐mapping studies we know that in many contexts neurons are born before glia, and that glia are attracted by and migrate towards differentiating neurons (Dearborn and Kunes, 2004), often receiving proliferative cues from them. Our data extend this theme by suggesting that the morphological differentiation of glia also occurs in response to, and induced by, neurons. Once installed, glia reciprocally regulate neuronal cell number by controlling neuronal stem cell proliferation and neuronal connectivity by controlling axon pruning and synapse formation.

In sum, our investigation of the glia in the adult nervous system has shown that glia are present in every part of the nervous system, forming a contiguous cellular network that covers and interweaves with the existing neuronal networks. Recent insights into the role of glia in the mature nervous system suggest complex homeostatic mechanisms that detect stress in neurons, resulting in prodegenerative signals from the glia to the neurons. Glia also influence neuronal performance by secretion of neuromodulators that influence synaptic activity, in addition to clearance of neurotransmitters and restoring the ionic milieu. The distinct morphologies and localization of the five generic glial subtypes, together with the fact that they are in contact with functionally distinct neuronal compartments, raises the question of whether the glial subtypes also perform distinct tasks. Our study provides the toolbox necessary to characterize the role of glia in the adult nervous system by a combination of transcriptome profiling and functional genomics, and thus determine directly whether the glial subtypes indeed have distinct physiological functions.

## Author Contributions

M.C.K. and U.G. conceived and designed the experiments. M.C.K. and S.B. performed the experiments. M.C.K. and C.J. analyzed the data. G.M.R. and U.G. wrote the article.

## Competing Interests

The authors declare no competing financial interests.

## Supporting information

Supporting Information

Supporting Information

Supporting Information

Supporting Information

Supporting Information

Supporting Information

Supporting Information

Supporting Information

Supporting Information

Supporting Information

Supporting Information

Supporting Information

Supporting Information

Supporting Information
