# Supplementary figures and images for: The glia of the adult Drosophila nervous system
Source: Glia. 2017 Jan 30;65(4):606–38. doi: 10.1002/glia.23115 (PMC5324652; doi:10.1002/glia.23115)

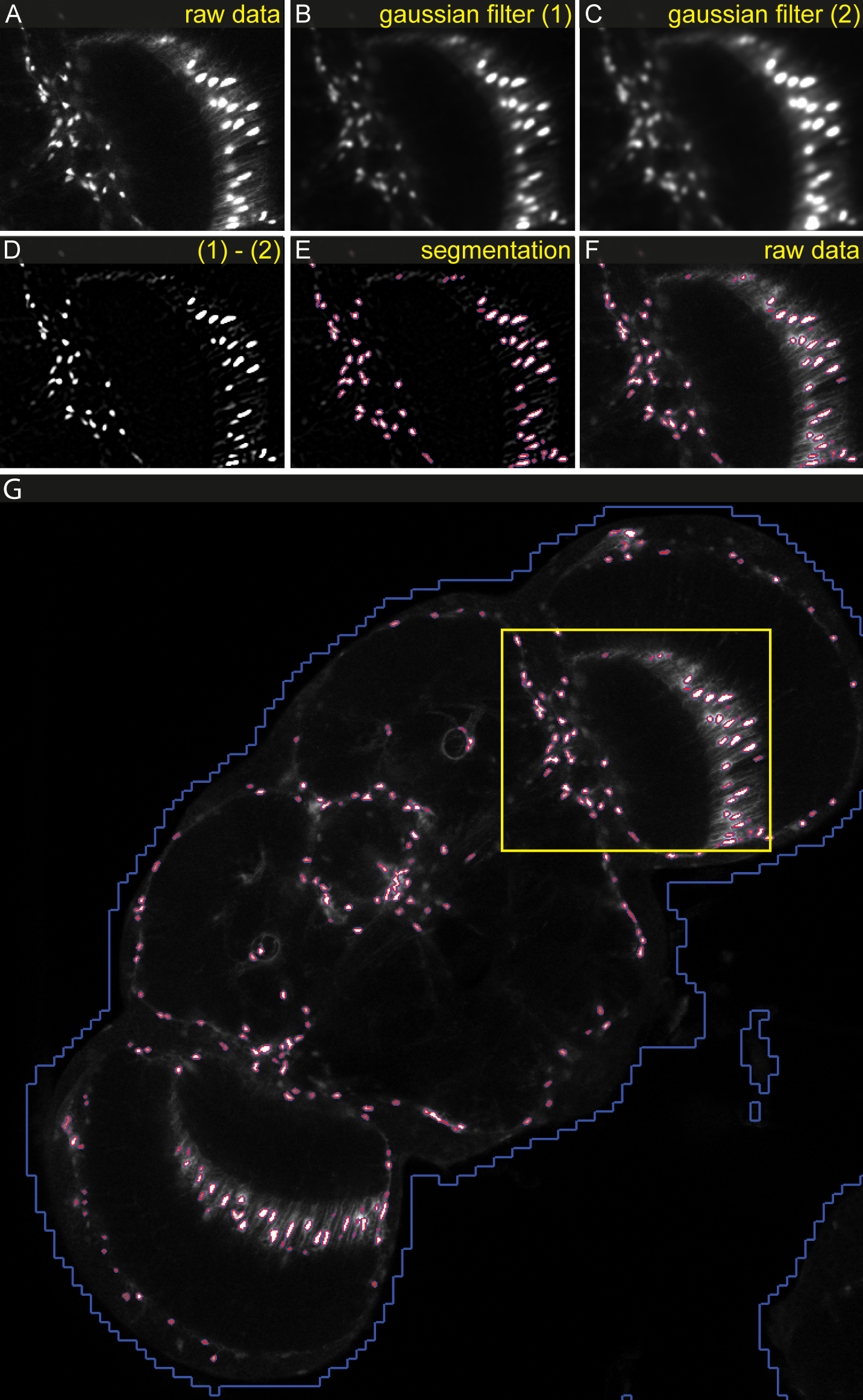

Supplement: Supplementary file 1 — Supporting Information [file GLIA-65-606-s001.tif]

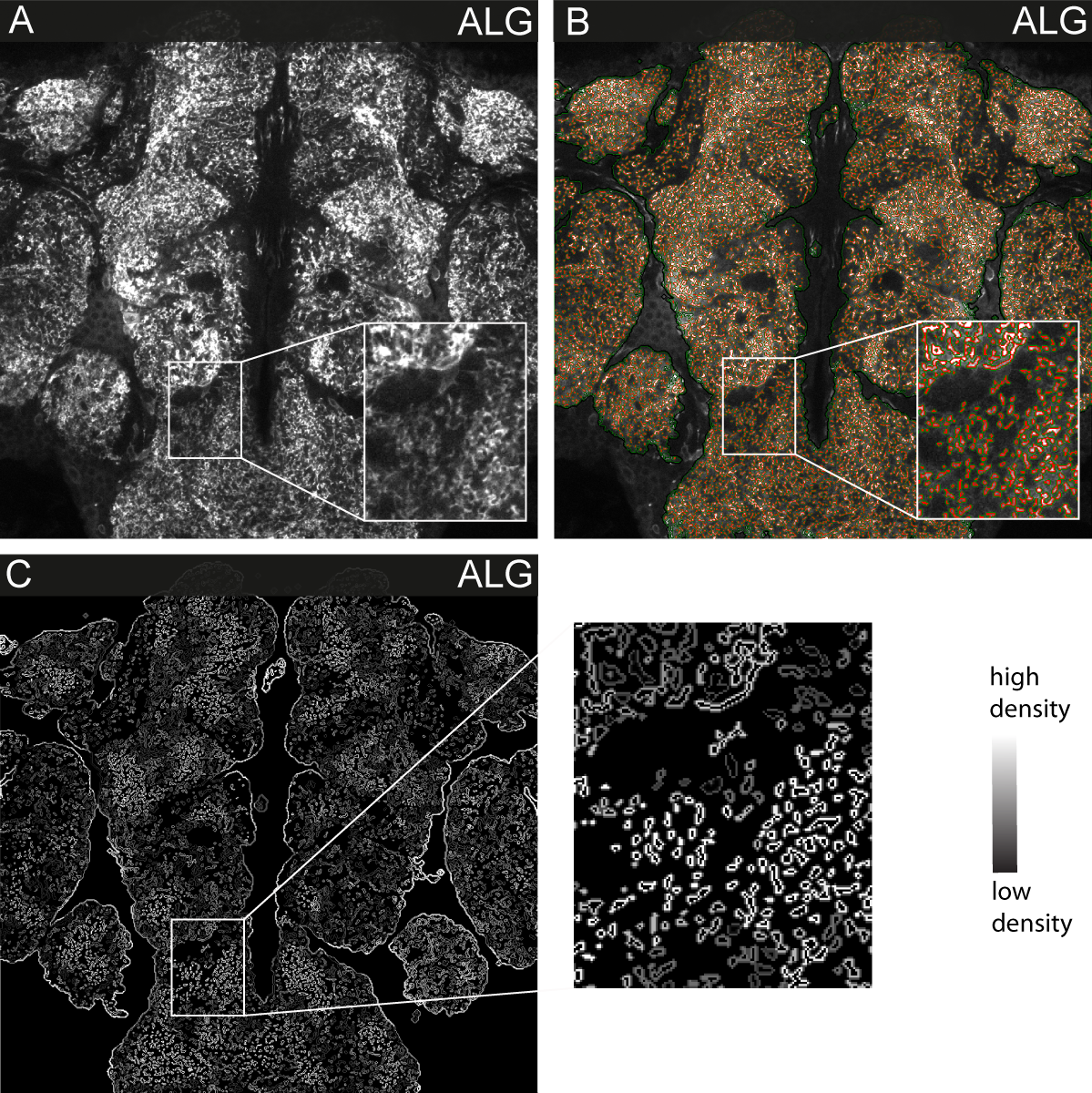

Supplement: Supplementary file 3 — Supporting Information [file GLIA-65-606-s003.tif]

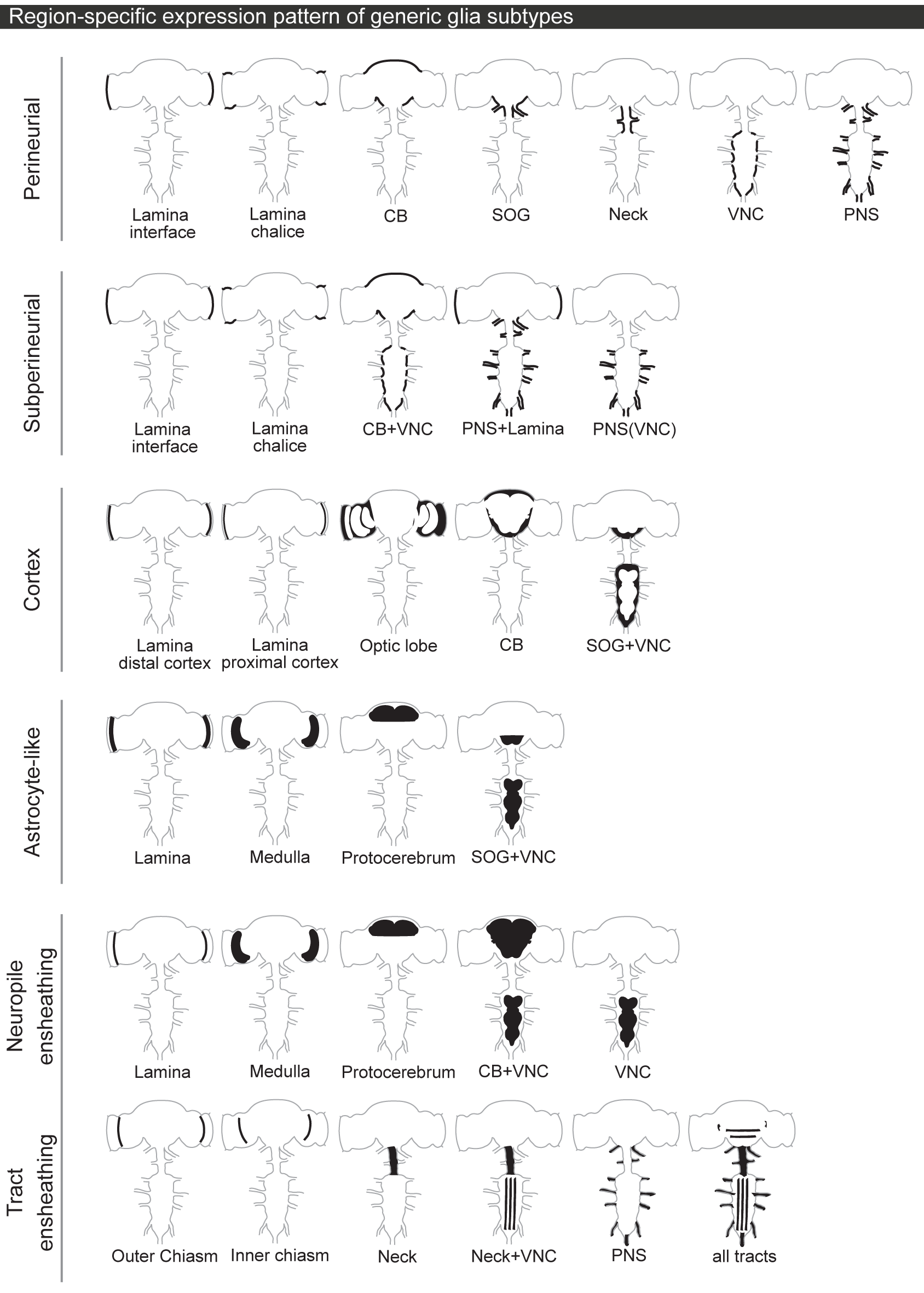

Supplement: Supplementary file 5 — Supporting Information [file GLIA-65-606-s005.tif]

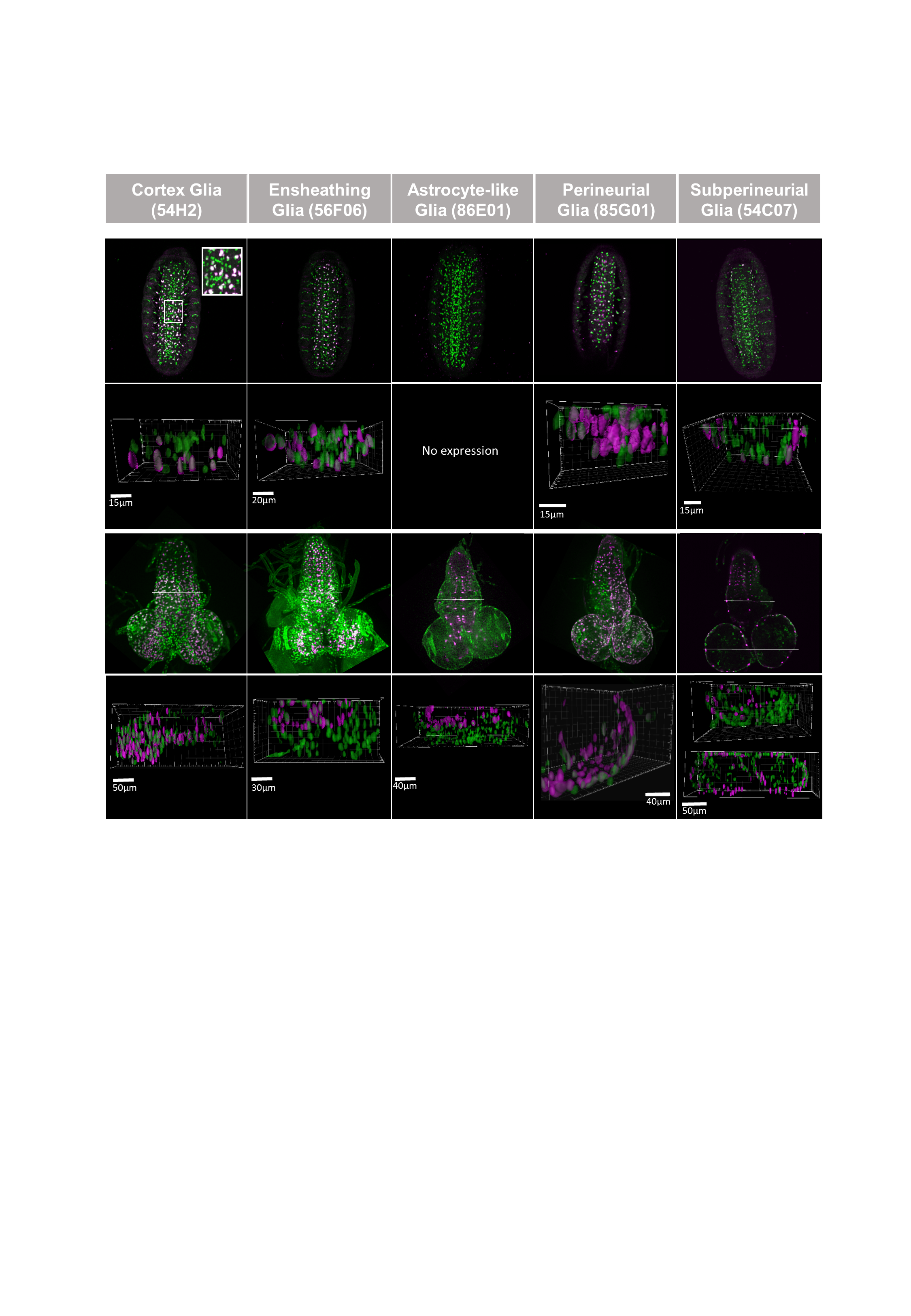

Supplement: Supplementary file 7 — Supporting Information [file GLIA-65-606-s007.tif]

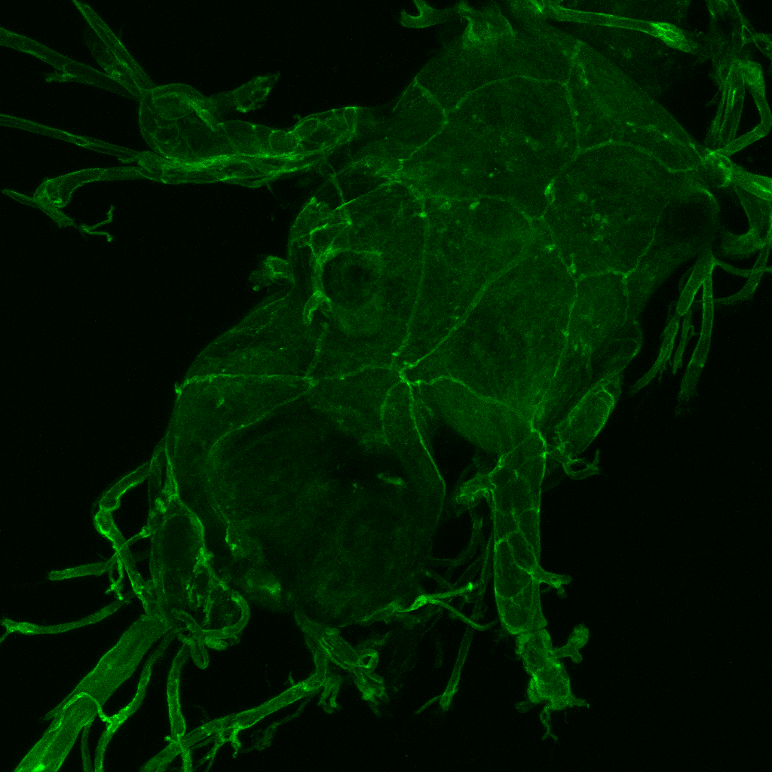

Supplement: Supplementary file 11 — Supporting Information [file GLIA-65-606-s011.tif]
