# Supplementary material for: The glia of the adult Drosophila nervous system
Source: Glia. 2017 Jan 30;65(4):606–38. doi: 10.1002/glia.23115 (PMC5324652; doi:10.1002/glia.23115)
Supplement: Supplementary file 2 — Supporting Information [file GLIA-65-606-s002.doc]

**Supplemental Figure 1: Automated counting of immune histochemically labeled glial nuclei.**

Confocal stacks of brains and ventral nerve chord, in which glial cell bodies were labeled using a nuclear GFP reporter (example here: 28A04-Gal4; UAS-nlsGFP) and REPO and neuronal cell bodies were co-labeled with ELAV, were processed as follows: The contours of the brain were identified based on the ELAV signal in channel one, the entire set of glial cell bodies was identified based on the REPO signal in channel two, subtype specific glial cell bodies were identified based on the GFP signal in channel three. Counting was carried out in 3D, however, in order to avoid over-counting, cell bodies were only counted in the z-layer with the highest density. To detect cell bodies, we applied the following strategy. In the raw images (**A**), which have a size of 1024x1024 pixels with a resolution of 692 nm per pixel in xy and a z slice of 1 µm, the signal intensity of GFP- and REPO-labeled nuclei bodies is high, but the considerable variation in background intensity makes a precise identification of cells difficult. Therefore, using a global threshold does not produce good segmentation. Instead, we developed a strategy based on background reduction. As a first step, we applied a 3D-Gaussian filter with a kernel size of 5x5x3 pixels (**B,** Gaussian filter (1)), then applied a second 3D-Gaussian filter, again with a kernel size of 5x5x3 pixels (**C**, Gaussian Filter (2)), and then subtracted **C** from **B**, which resulted in the background subtracted image shown in **D**. As a last step, we applied a global threshold and carried out segmentation using an algorithm implemented in the Definiens XD 2.0 software platform, as shown in **E**. Briefly, the Multi-Threshold Segmentation algorithm splits the image domain and classifies the resulting image objects based on a defined pixel value threshold. **F** shows the segmentation patterns (magenta lines) superimposed on the raw data. Despite strong background heterogeneities, as seen on the right, cells are readily identified. **G**. The 3D segmentation patterns are displayed for an exemplary confocal plane of an entire *Drosophila* brain. Finally, the data were exported and statistically evaluated in Microsoft Excel and GraphPath Prism.
