# Supplementary material for: The glia of the adult Drosophila nervous system
Source: Glia. 2017 Jan 30;65(4):606–38. doi: 10.1002/glia.23115 (PMC5324652; doi:10.1002/glia.23115)
Supplement: Supplementary file 4 — Supporting Information [file GLIA-65-606-s004.doc]

**Supplemental Figure 2: Automated analysis of the astrocyte-like glia-processes density.**

The structural density of astrocyte-like glia processes (ALG channel, **A**) and synapses (NC82 channel, **B**) were determined and correlated using a custom Definiens XD 2.0 script. The analysis strategy is illustrated for the ALG channel. First, the ALG processes were segmented similarly to the procedure described in Supplemental Figure 2: First, we applied a 3D-Gaussian filter with a kernel size of 5x5x3 pixels (Supp. Fig. 2**B**), then a second 3D-Gaussian filter, again with a kernel size of 5x5x3 pixels (Supp. Fig. 2**C**), and then subtracted **C** from **B**, which resulted in a background subtracted image (Supp. Fig. 2**D**). As a last step, we applied a global threshold and carried out segmentation using an algorithm implemented in the Definiens XD 2.0 software platform. The Definiens Multi-Threshold Segmentation algorithm splits the image domain and classifies the resulting image objects based on a defined pixel value threshold. **C** The segmented patterns of the brain (green) and of the ALG processes (red) are superimposed. **D.** The ALG process density was determined as follows. For each individual ALG process the total area of surrounding ALG processes was used as a measurement of the local process density. For each single image object of interest, we computed the total area in pixels of the neighboring objects present in a radius of 30 pixels around the center of mass of the image object of interest. The image objects were then color-coded according to their density, revealing regions with different ALG coverage. Finally, the procedure was reiterated with the NC82 channel. Subsequently, the two channels were correlated as shown in Figure 5C.3. **E.** To determine the average density levels as well as variation in density within previously defined brain regions (**F**, Insect Brain Name Working Group), we manually looked up the densities in every major brain region.
