# Supplementary material for: The glia of the adult Drosophila nervous system
Source: Glia. 2017 Jan 30;65(4):606–38. doi: 10.1002/glia.23115 (PMC5324652; doi:10.1002/glia.23115)
Supplement: Supplementary file 6 — Supporting Information [file GLIA-65-606-s006.doc]

**Supplemental Figure 3: Illustration of the different region-specific expression patterns within each generic subclass.**

For the different generic subtypes of glial cells, different region specific driver lines were found and are schematically illustrated here. The corresponding driver lines are listed in Supplemental Table 2.
