# Supplementary material for: The glia of the adult Drosophila nervous system
Source: Glia. 2017 Jan 30;65(4):606–38. doi: 10.1002/glia.23115 (PMC5324652; doi:10.1002/glia.23115)
Supplement: Supplementary file 8 — Supporting Information [file GLIA-65-606-s008.doc]

**Supplemental Figure 4: Embryonic and larval expression of main Gal4 lines.**

The expression of the main subtype-specific glial drivers is characterized in embryo and larvae (repo in green, driver in magenta); depending on the line, maximum density projection, single section or 3D cross section are shown. The cortex glial driver is expressed in cortex glia in embryo and larva; the ensheathing glial driver is expressed in ensheathing glia in embryo and larva; the astrocyte-like glial driver is not expressed in the embryo, where this cell-type is absent, but is expressed in astrocyte-like glia in larvae; the perineurial glial driver is expressed ectopically in the embryo but is expressed in perineurial glia in the larva; the subperineurial glial driver is expressed in subperineurial glia in embryo and larva. Strong ectopic expression is found in the perineurial glial driver in the midgut of stage 16 embryos and larvae; very weak ectopic expression in the larval midgut is found in the perineurial glial driver and in the subperineurial driver.
