# Supplementary material for: The glia of the adult Drosophila nervous system
Source: Glia. 2017 Jan 30;65(4):606–38. doi: 10.1002/glia.23115 (PMC5324652; doi:10.1002/glia.23115)
Supplement: Supplementary file 10 — Supporting Information [file GLIA-65-606-s010.doc]

**Supplemental Figure 5: Strength comparison of main Gal4 lines.**

The glial-subtype specific Gal4 lines, repoGal4 in the locus and an 8 kb repo enhancer on the X are driving UAS-nucCherry and are imaged live (n=6-10; error bars = sem). The cortex glial (54H02), the ensheathing glial (56F03), the astrocyte-like glial (86E01), the perineurial glial (85G01) and the subperineurial glial (54C07) drivers are all expressed at higher levels than the *repo-Gal4* drivers.
