# Supplementary material for: The glia of the adult Drosophila nervous system
Source: Glia. 2017 Jan 30;65(4):606–38. doi: 10.1002/glia.23115 (PMC5324652; doi:10.1002/glia.23115)
Supplement: Supplementary file 12 — Supporting Information [file GLIA-65-606-s012.doc]

**Supplemental Figure 6: Nrg::GFP in peripheral nerves.**

Subperineurial marker Nrg::GFP in ventral nerve chord and peripheral nerves. In larger nerves, multiple subperineurial cells build the circumference; in smaller nerves, two cells are sufficient to build the circumference.
