# Supplementary material for: The glia of the adult Drosophila nervous system
Source: Glia. 2017 Jan 30;65(4):606–38. doi: 10.1002/glia.23115 (PMC5324652; doi:10.1002/glia.23115)
Supplement: Supplementary file 13 — Supporting Information [file GLIA-65-606-s013.doc]

**Supplemental Table 1: Summary of GAL4 drivers with region-specific expression**

**in generic glial subtypes**

| **Subtype** | **Region-specific expression** | **Remarks** | **GAL4** | **Alternative** |
| --- | --- | --- | --- | --- |
| **Perineurial glia** | Lamina | Lamina interface perineurial = fenestrated glia | R47G01 | R19D10 |
|  | Lamina | Lamina chalice perineurial glia | R10C12 | R27H11 |
|  | Central brain | Only suboesophageal ganglion | R40A03 |  |
|  | Ventral nerve chord |  | R38E12 |  |
|  | Neck connective |  | R60B10 |  |
|  | Brain and ventral nerve chord | No expression in periphery | R94A04 | R85G04 |
|  | Central brain | Only ventral parts | R86C05 |  |
|  | Brain and ventral nerve chord | Only peripheral nerves | R79H10 |  |
| **Subperineurial glia** | Lamina | Lamina interface subperineurial = pseudocartridge glia | R50A12 |  |
|  | Lamina | Lamina chalice subperineurial glia | (R24A11) |  |
|  | Peripheral nerves | Co-expression in Lamina interface subperineurial glia | R37G04 | R31H12 |
|  | Brain and ventral nerve chord | No expression in periphery | R94B06 | R59F06 |
|  | Ventral nerve chord | No expression in periphery | R88A04 |  |
| **Cortex glia** | Lamina | Distal cortex = satellite glia | R53B07 |  |
|  | Lamina | Proximal cortex = satellite glia | R44B12 |  |
|  | Optic Lobe | Only medulla and lobula complex | R65B12 |  |
|  | Central brain | Mosaic co-expression in other glia | R09F07 |  |
|  | Central brain and ventral nerve chord | Only suboesophageal ganglion,  co-expression in astrocyte-like glia | R54D10 |  |
| **Neuropile**  **ensheathing**  **glia** | Lamina | Lamina ensheathing = marginal glia | R35E04 | R19C02 |
|  | Medulla |  | R73B10 | R41G04 |
|  | Central brain and Lamina | Only protocerebrum | R54E05 |  |
|  | Brain and ventral nerve chord |  | R56F03 | R15B05 |
|  | Brain and ventral nerve chord | No expression in protocerebrum | R83E12 | (R28A04) |
|  | Ventral nerve chord | Mosaic expression | R69G05 |  |
| **Tract ensheathing glia** | Brain and ventral nerve chord |  | R75H03 | R93H03 |
|  | Brain and ventral nerve chord | Expression only in peripheral nerves | R43H01 |  |
|  | Neck connective |  | R74E08 |  |
|  | Nerve exits of brain and ventral nerve chord | Interface peripheral nerves with neuropile region,  co-expression in optic lobe neurons | R95B11 |  |
|  | Ventral nerve chord | Mosaic expression in neuropile ensheathing glia | R69G05 |  |
| **Astrocyte-like glia** | Lamina | Lamina astrocyte-like = epithelial glia | R55B03 |  |
|  | Medulla |  | R31E10 | R84B11 |
|  | Central brain | Mosaic expression in protocerebrum | R81A12 |  |
|  | Brain and ventral nerve chord | Mosaic expression | R77B12 |  |
|  | Central brain and ventral nerve chord | Only suboesophageal ganglion,  co-expression in cortex glia | R54D10 |  |
|  | Brain and ventral nerve chord | Mosaic expression in lamina | R86E01 | R75B11  R25H07 |
|  | Central brain | Only AMMC,  co-expression in ensheathing glia at nerve exits | R86F01 |  |
|  | Ventral nerve chord | Mosaic expression | R29A12 | R70G03 |
|  | Central brain and ventral nerve chord | Only suboesophageal ganglion | R78D09 |  |

Listed are the best region-specific drivers for each generic glial subtype. The brain regions that show expression (column 2), as well as additional features of the pattern (column 3) are named. Whenever available, alternative drivers are provided (column 5). Additional region-specific driver lines can be found in the annotation data, but may represent drivers with neuronal co-expression and/or mosaic/weak expression in the glia.
