# Supplementary material for: The glia of the adult Drosophila nervous system
Source: Glia. 2017 Jan 30;65(4):606–38. doi: 10.1002/glia.23115 (PMC5324652; doi:10.1002/glia.23115)
Supplement: Supplementary file 14 — Supporting Information [file GLIA-65-606-s014.doc]

Supplemental Table 2: Characterization of optic lobe specific glia - expression pattern and cell number.

| **Driver** | **Generic subtype** | **Region-specific subtype** | **alternative drivers** | **Cell count (specific)** | **+/-**  **(SEM)** |
| --- | --- | --- | --- | --- | --- |
| R47G01 | Perineurial | Lamina interface perineurial = fenestrated glia | --- | --- | --- |
| R27H11 | Perineurial | Lamina chalice perineurial glia | --- | --- | --- |
| R50A12 | Subperineurial | Lamina interface subperineurial = pseudocartridge glia | --- | 95  (n= 2) |  |
| R53B07 | Cortex | Lamina distal cortex = satellite glia | --- | 358 | 56.5  (n=9) |
| R46H12 | Cortex | Lamina proximal cortex = satellite glia | --- | 255 | 12.46  (n=8) |
| R55B03 | Astrocyte-like | Lamina astrocyte-like = epithelial glia | --- | 475 | 14.3  (n=14) |
| R35E04 | Neuropile-ensheathing | Lamina ensheathing = marginal glia | --- | 109 | 4.9  (n=12) |
| R53H12 | Tract-ensheathing | Outer chiasm glia | 9G07-Gal4 (specific) | 48 | 2.39  (n=13) |
| R31E10 | Astrocyte-like | Medulla astrocyte-like glia | --- | 371 | 32.8  (n=18) |
| R73B10 | Neuropile-ensheathing | Medulla ensheathing glia | --- | 345 | 7.9  (n=8) |
| R53H12 | Neuropile-ensheathing | All chiasm glia | --- | 43 | 2  (n=4) |
|  |  |  |  |  |  |
|  |  |  |  |  |  |

Listed are the best drivers with optic lobe-specific glial expression. Each generic glial subtype (column 2) is represented by a line with optic lobe-specific expression (column 3). When available, drivers with comparable quality are provided (column 4). For each specific driver, the number of cells (column 5), the standard error of the mean and the number of optic lobes counted (column 6) are listed. The methods used for cell counting are described in Suppl. Fig. 2.
